# Increased PRSS56 expression is a causal factor and therapeutic target for human axial high myopia

**DOI:** 10.1038/s41422-026-01241-9

**Published:** 2026-04-01

**Authors:** Boxuan Wu, Weijia Zeng, Kefu Tang, Jiawei Xiong, Xiaofen Mo, Qing Fu, Dan Fu, Renyuan Chu, Guoli Zhao, Lei Lu, Zhongfeng Wang, Lingqian Wu, Zhiqiang Yu, Xiangyu Zhou, Hongyan Wang

**Affiliations:** 1 Obstetrics and Gynecology Hospital, State Key Laboratory of Genetic Engineering, Fudan University, Shanghai, China; 2 Shanghai Key Laboratory of Metabolic Remodeling and Health, Institute of Metabolism and Integrative Biology, Fudan University, Shanghai, China; 3 Eye and ENT Hospital of Fudan University, NHC Key Laboratory of Myopia, Shanghai, China; 4 Department of Ophthalmology, Huashan Hospital of Fudan University, Shanghai, China; 5 State Key Laboratory of Medical Neurobiology and MOE Frontiers Center for Brain Science, Fudan University, Shanghai, China; 6 The Center for Medical Genetics, Hunan Key Laboratory of Medical Genetics & Hunan Key Laboratory of Animal Models for Human Diseases, School of Life Sciences, Central South University, Changsha, Hunan China; 7 Children’s Hospital of Fudan University, Shanghai, China

**Keywords:** Molecular biology, Developmental biology

## Abstract

High myopia (HM), characterized by significant ocular axial length elongation, affects hundreds of millions of people and is often inherited, particularly in cases that develop during childhood or adolescence. Although numerous myopia loci (MYP) have been identified, most causative genes remain undefined. Here, we analyzed two large HM pedigrees and refined the critical region through haplotype linkage analysis to a 3.9-Mb interval on 2q37.1, which was previously reported as MYP12 with an unknown pathogenic gene. Whole-genome sequencing identified the noncoding promoter variants c.-187G>T and c.-187G>C in *PRSS56*, encoding a trypsin-like serine protease, which exclusively co-segregated with all affected members in both pedigrees. Compared with matched controls, increased PRSS56 expression was observed in both patient-derived iPSCs carrying c.-187G>T and knock-in mice (c.-155G>T, corresponding to human c.-187G>T) that faithfully recapitulate myopia phenotypes. Noncoding *PRSS56* variants promote self-expression via enhanced binding to the transcription factor EGR1, as confirmed by dual-luciferase assays. Notably, we demonstrated that higher PRSS56 levels directly increase ocular axial length in a dose- and activity-dependent manner in multiple transgenic mouse models. Guinea pig myopia models consistently exhibited high *Prss56* expression, and short-wave light exposure reduced *Prss56* mRNA levels and attenuated further axial elongation. Mechanistically, higher PRSS56 expression was associated with reduced abundance of myosin-4 in the sclera and with molecular signatures of scleral remodeling, which were in turn correlated with axial elongation. In conclusion, our findings provide strong genetic and functional evidence for the pathogenic role of noncoding *PRSS56* variants in HM and highlight PRSS56 as a promising therapeutic target for juvenile HM.

## Introduction

Myopia is one of the most prevalent ocular disorders globally, with a particularly high occurrence rate of 40%–70% in East Asia.^1–3^ In individuals with myopia, light is focused in front of the retina. The etiology of myopia is multifactorial, and environmental influences, such as extensive near work, higher intelligence, and limited outdoor exposure, are associated with increased myopia risk.^4,5^ Although myopia is common, high myopia (HM) is rarer and more severe, characterized by an ocular axial length greater than 26 mm or a refractive error greater than −6.00 diopters (D).^6,7^ Genetic factors have been reported to play a key role in HM, especially in early-onset cases (before the age of 6). Twin studies have indicated a high heritability for HM, with estimates ranging from 0.50 to 0.96.^8^ Large-scale genome-wide association studies (GWAS) and meta-analyses have demonstrated that most refractive error and myopia risk at the population level are polygenic, implicating hundreds of common loci and multiple biological pathways.^9–12^ Complementing these polygenic findings, family-based linkage studies have mapped multiple myopia loci (MYP1–MYP28), but the causal genes for ~60% of inherited HM cases remain unidentified.^13,14^

Here, motivated by the value of rare, high-penetrance variants for revealing causal mechanisms, we investigated two large multigenerational Chinese families segregating early-onset high myopia (eoHM) and identified two rare, non-coding variants at the *PRSS56* locus (MYP12) that segregate with the disease. We performed genetic and functional analyses to assess the regulatory effects of these variants on *PRSS56* expression and their relationship to refractive error and axial elongation. MYP12 was first identified in 2005 as a novel locus on chromosome 2q37.1 in an autosomal dominant high-grade myopia family with 31 members, 14 of whom were affected. Despite mutation screening of two candidate genes, *SAG* and *DGKD*, no variants were found to segregate with affected status.^15^ Replication of the MYP12 locus was reported in three large Australian families with autosomal dominant myopia, in which 35 of 49 participants were affected.^16^ In 2009, another susceptibility locus for myopia was identified in the 2q37 region, which is adjacent to but does not fully overlap with MYP12.^17^ Although genetic evidence supporting an association between the 2q37 region and myopia has accumulated across different populations, the precise causative genes at the MYP12 locus remain to be determined. In 2013, a large-scale GWAS identified numerous new loci associated with myopia through meta-analyses involving over 40,000 participants.^18,19^ Notably, the identified SNP, rs1656404 (GRCh37, 2:233379941), was significantly associated with myopia and was located adjacent to *PRSS56*, a gene situated at the MYP12 locus (2q37.1). Since rs1656404 is located in an intergenic region, it was likely identified as a tag SNP for the pathogenic interval in GWAS analyses.

PRSS56 is a secreted protein that contains a peptidase S1 domain and exhibits trypsin-like serine protease activity. Previous studies have linked loss-of-function mutations in *PRSS56* to hyperopia and angle-closure glaucoma.^20–23^ The homozygous mutations p.Gln356Profs*152 and p.Pro599Ala were identified in two Tunisian families with posterior microphthalmia. The affected individuals had a reduced ocular axial length of less than 20 mm and high hyperopia. Consistent with these findings, the eyes of *Prss56* knockout (KO) mice had a shorter axial length than those of wild-type (WT) mice. Here, we report that non-coding variants of *PRSS56* promote self-expression, contributing to autosomal dominant HM. Different *PRSS56* variants that lead to opposite refractive outcomes indicate the critical role of PRSS56 dose or activity in the regulation of axial length, as well as in the transition from normal vision to refractive error. Using a variety of approaches, we found that increased PRSS56 expression directly promotes ocular axial elongation and contributes to axial HM. Interestingly, guinea pig models of myopia created by different methods consistently exhibited high *Prss56* expression, indicating that increased PRSS56 is both a marker of myopia and a causal factor. Our findings highlight PRSS56 as a promising therapeutic target for HM from childhood to adulthood. In practice, we showed that blue-light exposure significantly attenuated the progression of myopia by downregulating *Prss56* expression in pigmented guinea pigs.

## Results

### Novel non-coding *PRSS56* variants co-segregate with HM in two large families

**Fig. 1:**
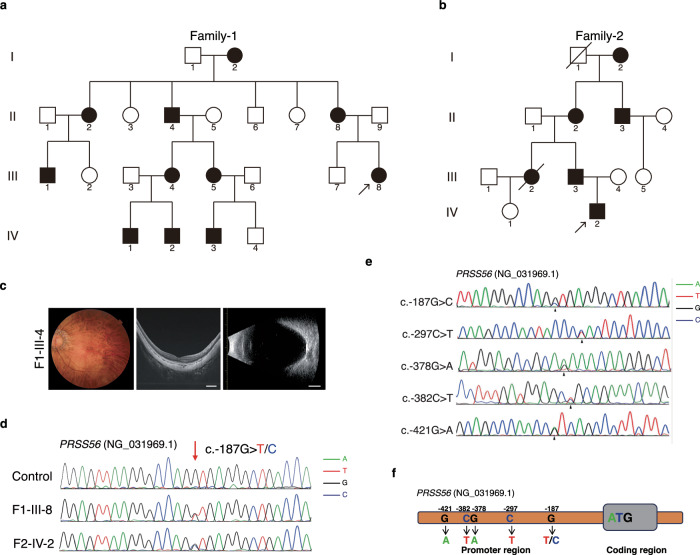
Novel non-coding variants in the *PRSS56* promoter were identified in two multigenerational HM families. **a** Pedigree of Family 1, with 11 affected (black) and 12 unaffected (white) individuals. Proband F1-Ⅲ-8 was a 6-year-old girl with severe myopic refractive error. **b** Pedigree of Family 2, with 6 affected and 4 unaffected individuals. Proband F2-Ⅳ-2 was a 4-year-old boy with severe myopic refractive error. **c** Optical examination of an affected individual (F1-Ⅲ-4) showed a leopard-like fundus, temporal papillae of the optic papilla, and macular degeneration (left). Optical coherence tomography indicated that the retinal thickness at the fovea was decreased (middle). Scale bar, 250 μm. B-scan ultrasonography showed extreme lengthening of the ocular axis and posterior staphyloma (right). Scale bar, 5 mm. **d** Sequencing confirmed the heterozygous non-coding variants in *PRSS56* (c.-187G>T and c.-187G>C) that segregated with disease in the families. The identified mutation is indicated by a red arrow. **e** Five rare, disease-specific heterozygous *PRSS56* variants (c.-187G>C, c.-297C>T, c.-378G>A, c.-382C>T, and c.-421G>A) identified among 236 sporadic HM cases were confirmed by Sanger sequencing. Variant c.-382C>T was observed in two cases. For **d** and **e**, nucleotide colors indicate adenine (A, green), thymine (T, red), guanine (G, black), and cytosine (C, blue). **f** *PRSS56* promoter variants identified in familial and sporadic cohorts.

We collected two large multigenerational Chinese families (F1 and F2) in which adolescent-onset HM segregated in an autosomal-dominant manner (Fig. 1a, b). The pedigrees included 17 affected individuals (11 in F1 and 6 in F2) who displayed a familial ocular phenotype of eoHM; many subsequently developed cataracts, peripheral retinal degeneration, and subluxated lenses as they aged and underwent posterior scleral reinforcement surgery (Fig. 1c; Supplementary information, Fig. S1a).

To identify the disease locus, we performed a multipoint non-parametric genome-wide linkage analysis of 21 individuals from F1. A maximum LOD score of 3.81 was obtained at rs12621510 (2:235051708) on chromosome 2q37.1, with a recombination fraction θ of 0.00. No other genomic intervals yielded an LOD score greater than 1.20 across the remaining 21 autosomes (Supplementary information, Fig. S1b). Haplotype analyses narrowed the interval to a 3.9-Mb region from rs17619600 (2:231976460) to rs2042831 (2:235857117) that encompassed the MYP12 locus (Supplementary information, Fig. S1c). In particular, rs1656404 (2:233379941) from a previous GWAS was also located in this critical interval.^18^

To identify the causative variants, we initially performed whole-exome sequencing to profile all genetic alterations within the interval. However, no candidate variants in the coding sequences of the identified genes met both the minor allele frequency (MAF < 0.01) and co-segregation requirements. Significant chromosomal structural variants and pathogenic copy number variations (CNVs) were also ruled out through ExomeDepth analysis and array-based comparative genomic hybridization (aCGH).

Since strong genetic evidence for a relationship between 2q37.1 and HM was obtained in our work and in previous studies of different ethnicities, we performed genome-wide sequencing (WGS) and identified two novel heterozygous non-coding variants (c.-187G>T and c.-187G>C) (2:233385122) in the two families, respectively (Fig. 1d; Supplementary information, Table S1). Notably, the two non-coding variants were the only candidate variants that simultaneously met both the minor allele frequency criterion (MAF < 0.01) in gnomAD and co-segregated with all 17 affected individuals from the two families within the critical 3.9-Mb interval. Both variants have no recorded frequency in the gnomAD database (0/152,330) and were absent in 653 ethnically matched normal controls. Although the base substitution at site −187 differed between F1 (G>T) and F2 (G>C), the presence of recurrent variants at the same locus provides strong evidence for the pathogenicity of non-coding variants in HM.

We next performed variant screening of the 603-bp upstream region of *PRSS56* by Sanger sequencing of 236 sporadic early-onset HM cases and identified five disease-specific variants of *PRSS56* in six sporadic cases (c.-187G>C, c.-297C>T, c.-378G>A, c.-382C>T, and c.-421G>A) (Fig. 1e, f). Four variants (c.-187G>C, c.-297C>T, c.-378G>A, and c.-382C>T) had no frequency records in gnomAD. The recurrent *PRSS56* variant c.-187G>C in a sporadic HM case once again highlighted its role in genetic pathogenicity. Analysis of evolutionary conservation suggested that most of these non-coding *PRSS56* variants are conserved across mammalian species with advanced vision (Supplementary information, Fig. S2a–c).

### Non-coding *PRSS56* variants enhance EGR1 binding and promote self-expression

**Fig. 2:**
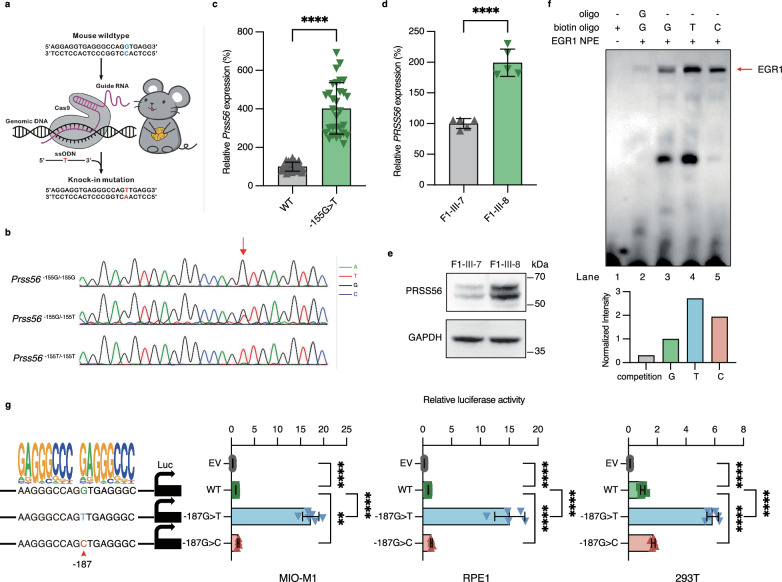
c.-187G variants significantly promote self-expression by enhancing EGR1 binding. **a** Schematic of the *Prss56* G-to-T single-base KI mouse model generated using CRISPR/Cas9 sgRNA. **b** The mutation in the *Prss56* (c.-155G>T) KI mice was confirmed by Sanger sequencing. The c.-155 site is indicated by a red arrow. **c** Increased *Prss56* mRNA expression in the retina of *Prss56*^*−155T/−155T*^ mice at postnatal day 1; *n* = 30 mice per genotype. **d** Increased *PRSS56* mRNA levels in iPSCs derived from peripheral blood of an affected individual carrying c.-187G>T and her unaffected sibling; *n* = 5 technical replicates per group. **e** Increased PRSS56 protein expression in retinal organoids derived from HM patient-derived iPSCs. **f** An EMSA demonstrates robust binding of EGR1 to the c.-187 PRSS56 variants. The red arrow denotes the specific protein–DNA complex, and the corresponding densitometric quantification is shown. **g** Increased luciferase expression driven by c.-187 *PRSS56* variants in two retina-derived lines and non-retinal cell lines; *n* = 5–6 technical replicates per group. The c.-187 site is indicated by a red arrowhead.

The novel variant c.-187G>T/C is located 55 bases downstream of a TATA box, suggesting a potential regulatory role in *PRSS56* expression. We hypothesized that the identified non-coding variants that promote *PRSS56* expression might induce myopia, as individuals with loss-of-function mutations in the *PRSS56* coding region typically exhibit hyperopia. To test this possibility, we generated a murine knock-in (KI) model (mouse c.-155G>T, corresponding to human c.-187G>T) using CRISPR/Cas9 technology and determined the mouse genotypes by PCR and Sanger sequencing (Fig. 2a, b). Compared with WT littermates, *Prss56*^*−155T/−155T*^ mice showed significantly higher *Prss56* expression in eye tissues at postnatal day 1 (Fig. 2c). We also generated induced pluripotent stem cells (iPSCs) from an HM patient (III-8) and her unaffected sibling (III-7) in F1 (Supplementary information, Fig. S3a, b) and found significantly higher *PRSS56* mRNA levels in the patient-derived iPSCs (Fig. 2d). Following the established standard protocol, we next generated retinal organoids from the patient and control lines (Supplementary information, Fig. S3c, d). By day 48, well-formed organoids were evident, and western blotting confirmed that PRSS56 protein expression was markedly higher in the patient-derived organoids than in the healthy controls (Fig. 2e).

To investigate the mechanisms underlying increased *PRSS56* expression, we performed motif scanning on a 200-bp sequence surrounding the c.-187 mutation site using MEME software. Enrichment analysis suggested that the mutant alleles may enhance the binding of the EGR1 (early growth response protein 1) transcription factor to the *PRSS56* promoter region. The c.-187 nucleotide resides within an EGR1 consensus motif and lies ~80 bp upstream of the annotated *PRSS56* transcription start site; an additional nearby EGR1 motif is immediately adjacent to this position. Electrophoretic mobility shift assays (EMSAs) were performed to evaluate the binding of EGR1 to *PRSS56* c.-187 promoter probes. First, to confirm the identity of the shifted complex, we carried out antibody supershift assays by adding an anti-EGR1 antibody, which produced a reproducible supershift of the principal shifted band and a concomitant reduction in the original complex (Supplementary information, Fig. S3e). Analysis of allele-specific binding revealed that EGR1 exhibited stronger DNA-binding affinity for probes containing the mutant alleles (−187T or −187C) compared with the WT allele (−187G) (Fig. 2f). To further confirm the interaction specificity, we performed competition assays. Increasing concentrations of an unlabeled specific competitor probe progressively decreased the intensity of the shifted complex until it disappeared completely. By contrast, a scrambled-sequence competitor probe did not affect complex formation. These results confirm that the indicated band corresponds to the specific EGR1–biotinylated probe complex (Supplementary information, Fig. S3f). We next generated luciferase reporters containing a 603-bp fragment of the *PRSS56* proximal promoter to test whether EGR1 can modulate *PRSS56* promoter activity. Overexpression of EGR1 significantly increased the activity of the *PRSS56* promoter by ~10-fold across two retinal cell lines (MIO-M1 and RPE1) and the non-retinal HEK293T cell line. More importantly, introduction of both mutant alleles (−187 T and −187 C) resulted in significantly higher promoter activity than introduction of the WT allele (−187 G) upon co-transfection with EGR1 in both retinal and non-retinal cell lines (Fig. 2g). These results indicate that the *PRSS56* c.-187G>T/C variants enhance transcription by facilitating EGR1 binding. All HM-associated variants in the *PRSS56* promoter displayed significantly higher transcriptional activity than the WT, consistent with our hypothesis (Supplementary information, Fig. S3g).

### Increased *Prss56* expression is consistently correlated with ocular axial elongation

**Fig. 3:**
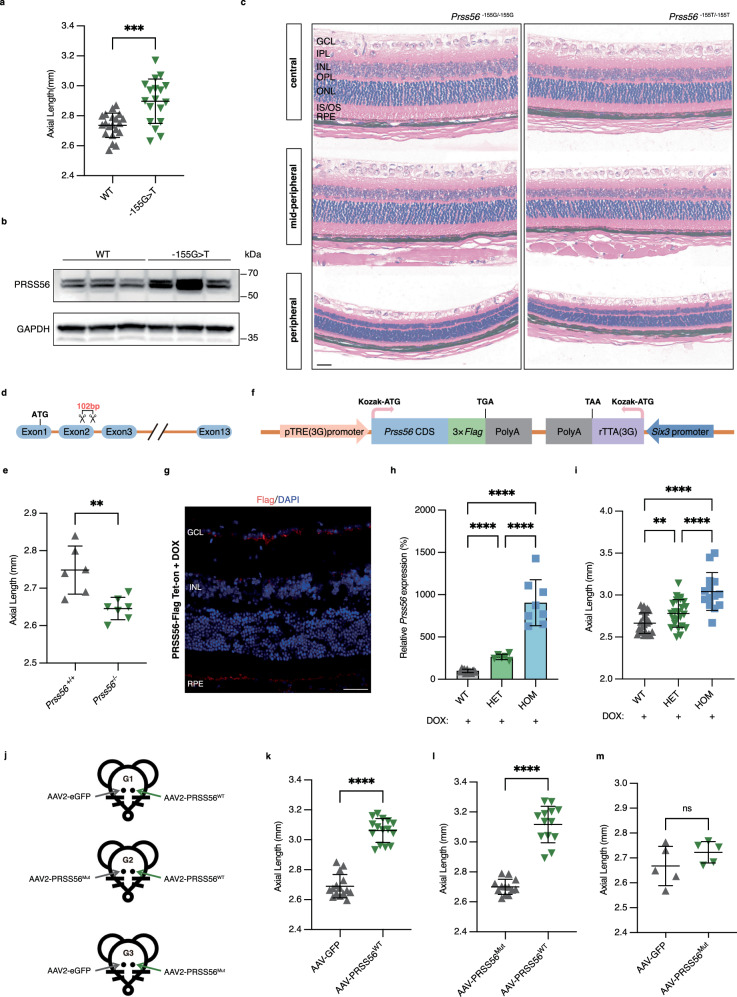
PRSS56 determines ocular axial length in a dosage- and activity-dependent manner. **a** *Prss56*^*−155T/−155T*^ mice have increased axial length at 4 weeks of age; *n* = 18–20 mice per genotype. **b** Increased PRSS56 protein expression in whole eyes of *Prss56*^*−155T/−155T*^ mice at 4 weeks of age; *n* = 3 mice per genotype. **c** The location of different cell types and H&E staining of the central, mid-peripheral, and peripheral retina at 4 weeks of age. The central, mid-peripheral, and peripheral retinal thicknesses of KI mice were lower than those of WT mice. Scale bar, 20 μm. **d** Schematic representation of *Prss56* KO mice generated using the CRISPR/Cas9 system. A deletion of 109 bp (red) spanning from exon 2 to the exon–intron junction region was identified using Sanger sequencing. **e** *Prss56*^*−/−*^ mice have decreased axial length at 4 weeks of age; *n* = 6–7 mice per genotype. **f** Schematic of the edited allele of PRSS56-Tet-on mice. **g** Immunofluorescence (IF) analysis localized DOX-induced *PRSS56* expression primarily to the retinal GCL, INL, and regions adjacent to the RPE. Scale bar, 20 μm. **h** Increased *Prss56* mRNA expression in the retina of PRSS56-Tet-on mice; *n* = 6–12 mice per genotype. **i** Axial lengths of eyes in PRSS56-Tet-on mice were related to *Prss56* expression levels in eyes; *n* = 14–24 mice per genotype. **j** Schematic of a mouse model in which AAV2-PRSS56^WT^, AAV2-eGFP, or AAV2-PRSS56^Mut^ was injected into the vitreous cavity. **k** Increased ocular axial lengths of eyes injected with AAV2-PRSS56^WT^ relative to controls injected with AAV2-eGFP; *n* = 15 mice. **l** Increased ocular axial lengths of eyes injected with AAV2-PRSS56^WT^ relative to eyes injected with AAV2-PRSS56^Mut^; *n* = 13 mice. **m** There were no significant differences in axial length between mice injected with AAV2-eGFP and AAV2-PRSS56^Mut^; *n* = 5 mice.

To test whether *Prss56*^*−155T/−155T*^ mutant mice exhibited increased axial length, we performed ocular biometry using A-scan ultrasonography (Supplementary information, Fig. S4a). We found that *Prss56*^*−155T/−155T*^ mutant mice developed normal eye structure and diameter, but the axial lengths of the KI mice (2.897 ± 0.1439 mm) were significantly longer than those of WT controls (2.737 ± 0.0792 mm) (Fig. 3a), consistent with the elevated PRSS56 protein expression in the KI mice (Fig. 3b). Histopathological examination and quantitative morphometry of central retinal sections revealed thinner retinas in the KI mice at 4 weeks of age (Fig. 3c; Supplementary information, Fig. S4b). These findings demonstrate that the *Prss56* KI mice faithfully recapitulate the elongated axial length phenotype observed in HM patients, supporting the pathogenicity of the *PRSS56* c.-187G>T variant.

As a complementary loss-of-function control, we generated a new *Prss56* KO mouse model (*Prss*56^*−*/*−*^, c.114_214del) (Fig. 3d; Supplementary information, Fig. S4c, d). These KO mice exhibited the expected opposite phenotype, with a significant reduction in axial length at postnatal week 4 (Fig. 3e), consistent with previous reports.^24,25^ Together, the reciprocal KI and KO results support a dosage-dependent influence of PRSS56 on ocular axial dimensions.

We next hypothesized that altered PRSS56 expression outside of the neonatal period may also influence ocular growth, and we generated a new transgenic mouse model using the Tet-on system to induce retina-specific PRSS56 expression (Fig. 3f; Supplementary information, Fig. S4e, f). We opted for the *Six3* promoter, as it drives expression confined to the retina in ocular tissues.^26^ Indeed, doxycycline (DOX) treatment induced PRSS56 expression in the retina, as expected, and IF showed PRSS56 localization in the ganglion cell layer (GCL), the inner nuclear layer (INL), and regions adjacent to the retinal pigment epithelium (RPE) (Fig. 3g). This distribution was consistent with secretion of PRSS56 toward both the vitreal and subretinal sides of the retina and raised the possibility that PRSS56 acts on multiple local substrates and fulfils diverse functions across retinal and peri-retinal compartments. We detected *Prss56* overexpression in the retina within 3 days after the initiation of DOX treatment in the drinking water (Fig. 3h). The lack of expanded PRSS56 expression in WT mice treated with DOX and in homozygous Tet-on mice without DOX treatment provided clear evidence that our system did not show leaky expression (Supplementary information, Fig. S4g). More importantly, DOX induction of *Prss56* produced dose-dependent increases in axial length (Fig. 3i). These findings support the view that PRSS56 upregulation is causally linked to axial elongation and therefore suggest PRSS56 as a potential therapeutic target for myopia prevention.

We considered that PRSS56, as a serine protease, exerts its biological function not only through its expression level but also through the regulation of its enzymatic activity. We therefore selectively infected the retina using WT AAV2-PRSS56^WT^ and catalytically inactivated AAV2-PRSS56^Mut(H149A)^ viruses to investigate the functional contribution of PRSS56 activity (Fig. 3j). The viruses were individually injected into the vitreous cavities of 1-day-old mice to generate a model of increased WT PRSS56 expression and a model of increased PRSS56 expression in which the enzyme activity center was inactivated (Supplementary information, Fig. S4h). The H149 residue, located at the enzymatic center of PRSS56, was mutated to alanine (A) to mimic inactivation of the protein. The expression efficiency of the AAV2 virus was assessed through IF and western blot analyses of the stretched retina at postnatal day 14 (Supplementary information, Fig. S4i, j). Spectral-domain optical coherence tomography (SD-OCT) suggested that ocular axial length in AAV2-PRSS56^WT^ mice was significantly greater than that of the contralateral eye injected with AAV2-PRSS56^Mut^ or AAV2-eGFP (Fig. 3k, l). By contrast, no significant difference in axial length was observed between mice injected with AAV2-PRSS56^Mut^ and those receiving empty AAV2-eGFP viruses (Fig. 3m).

Collectively, the concordant directionality across gain- and loss-of-function models, together with the activity dependence demonstrated by the catalytic mutant, supports a role for PRSS56 as a determinant of axial growth, with its increased expression promoting axial elongation.

### Mice with increased PRSS56 expression developed the same pathological ophthalmic phenotype as HM patients carrying *PRSS56* variants

**Fig. 4:**
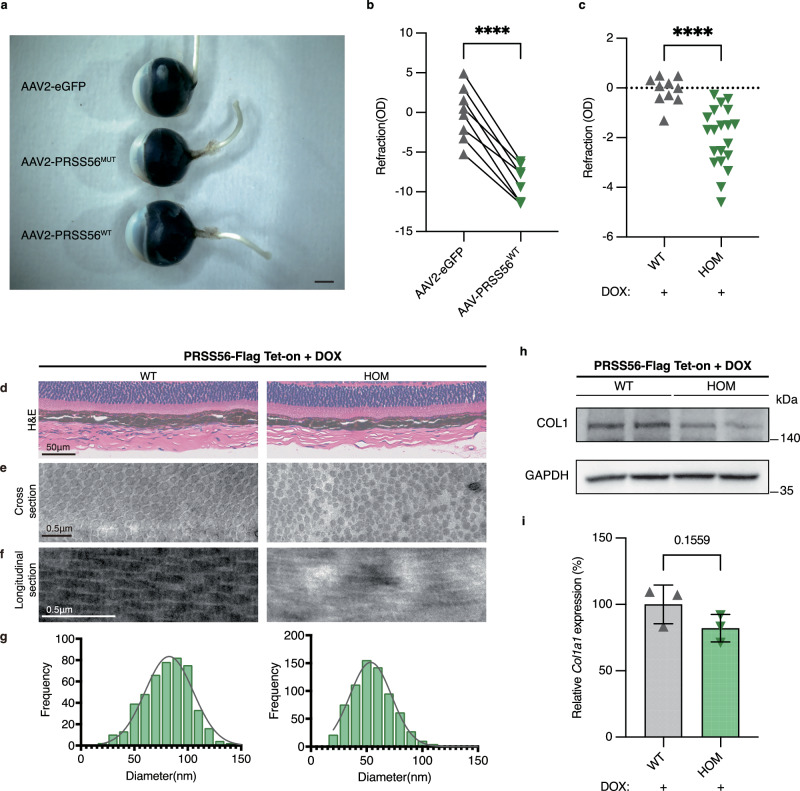
Morphological changes in the retina and sclera of PRSS56-overexpressing mice. **a** AAV2-PRSS56^WT^ injection increases eye size relative to that of AAV2-PRSS56^Mut^ or AAV2-eGFP controls. Scale bar, 1 mm. **b** Eyes injected with AAV2-PRSS56^WT^ were significantly more myopic than contralateral eyes injected with AAV2-eGFP; *n* = 8 eyes per condition. **c** Eccentric infrared photorefraction measurements show a significant myopic shift in DOX-treated Tet-on mice compared with controls; *n* = 10–20 mice per genotype. **d** H&E staining of the sclera revealed that DOX-induced Tet-on mice exhibited significant scleral thinning compared with WT controls. Scale bar, 50 μm. **e**–**g** Transmission electron microscopy of cross sections (**e**) and longitudinal sections (**f**) of the posterior sclera revealed heterogeneously sized collagen fibrils with irregular contours, enlarged interfibrillar spacing, and significantly reduced diameters (**g**) in DOX-induced Tet-on mice compared with WT controls. Scale bars, 0.5 μm. **h**, **i** Western blot (**h**) and qPCR (**i**) analyses revealed significantly reduced type Ⅰ collagen expression in scleral tissues of DOX-induced Tet-on mice compared with WT controls; *n* = 3 mice per genotype.

Consistent with the elongated axial length, the gross eyeball volume was significantly larger in mice injected with AAV2-PRSS56^WT^ than in those injected with AAV2-PRSS56^Mut^ or AAV2-eGFP, as confirmed by anatomical sampling (Fig. 4a). Eyes injected with AAV2-PRSS56^WT^ also exhibited a significantly altered refractive error compared with the contralateral eyes injected with AAV2-eGFP (Fig. 4b).

Refractive data from the Tet-on cohort showed that PRSS56 overexpression induced by DOX treatment led to a significant myopic shift (Fig. 4c). SD-OCT revealed no significant differences in the corneal radius of curvature or anterior lens position between PRSS56-overexpressing mice and controls (Supplementary information, Fig. S5a). Together with the increased axial length observed in this model (Fig. 3i), these findings indicate that Tet-on PRSS56 mice exhibit refractive and biometric characteristics consistent with axial myopia.

Hematoxylin and eosin (H&E) staining of eyeball sections from DOX-induced PRSS56-overexpressing Tet-on mice demonstrated a pronounced thinning of the neural retina compared with controls. Morphometric analysis confirmed a significant reduction in total retinal thickness in PRSS56-overexpressing eyes, with the greatest proportional loss observed in the INL and substantial decreases also apparent in the RPE (Supplementary information, Fig. S5b–d). The anatomical pattern of thinning was consistent with the involvement of Müller glia, cell types previously reported to express *PRSS56*, although the present histology alone does not provide definitive cell-type-specific evidence.^24^ Further targeted experiments are required to confirm whether Müller glia are the primary source mediating this effect. To further investigate the effect of PRSS56 on retinal structure, we examined the external limiting membrane (ELM) by immunofluorescent labeling of the tight junction protein ZO-1, which links Müller cell endfeet to photoreceptor inner segments.^27^ In PRSS56-overexpressing retina, ZO-1 immunoreactivity at the ELM was markedly diminished and patchy, and western blot analysis revealed a corresponding reduction in total ZO-1 protein (Supplementary information, Fig. S5e–g).

Given that scleral remodeling is the primary driver of myopia and strongly correlates with axial elongation, we assessed scleral collagen in Tet-on mice. Retinal overexpression of PRSS56 induced profound scleral abnormalities, including reduced collagen fiber diameter, disorganized fiber arrangement, and decreased overall collagen content (Fig. 4d–g). Quantitative PCR (qPCR) and western blotting further confirmed a significant downregulation of type I collagen, the principal structural protein of the sclera (Fig. 4h, i). Two PRSS56-overexpression mouse models created through AAV injection and the Tet-on system exhibited marked morphological changes in the retina and sclera, closely resembling the phenotype observed in KI mice and patients with *PRSS56* promoter mutations. These findings suggest that increased PRSS56 levels disrupt ocular development and contribute to myopia-related changes.

### PRSS56 overexpression is associated with reduced abundance of myosin-4 in sclera and ciliary-region tissues

**Fig. 5:**
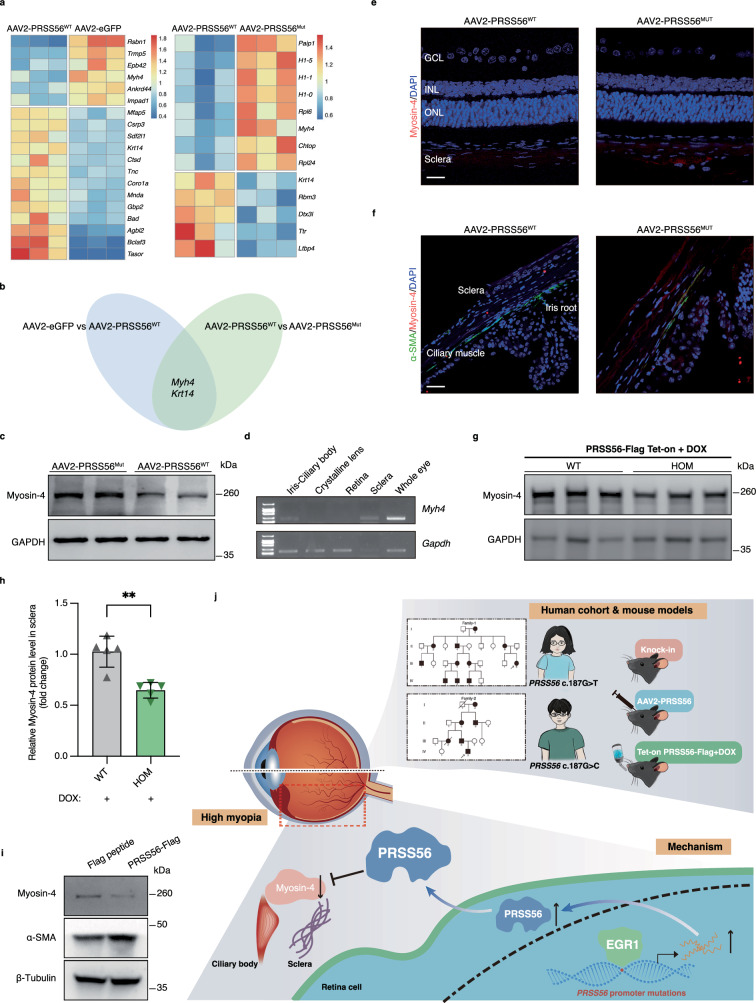
Overexpression of PRSS56 is associated with reduced myosin-4 abundance. **a** Heat-map representation of 19 and 13 significantly differentially expressed proteins (*P* < 0.05, fold change > 1.5, FDR < 1%) in groups 1 and 2, respectively. **b** Differential expression of *Myh4* and *Krt14* was identified in both groups. **c** Reduced myosin-4 expression in whole eyes injected with AAV2-PRSS56^WT^ shown by western blot. **d** Semi-qPCR analysis of *Myh4* transcripts in dissected ocular tissues. Samples included the isolated ciliary body, crystalline lens, neural retina, sclera, and whole adult eye. Data show detectable *Myh4* transcripts in the ciliary body and sclera preparations. **e** IF of the posterior sclera showed reduced myosin-4 localization in AAV2-PRSS56^WT^ mice compared with AAV2-PRSS56^Mut^ mice. Scale bar, 20 μm. **f** IF staining for α-SMA (ACTA2) and myosin-4 revealed decreased Myosin-4 signal in the ciliary body region of AAV2-PRSS56^WT^ mice compared with AAV2-PRSS56^Mut^ mice. Scale bar, 20 μm. **g** Reduced myosin-4 expression in scleral lysates from DOX-induced Tet-on mice and age-matched WT littermates shown by western blot. **h** Quantification of myosin-4 protein levels normalized to that of GAPDH; *n* = 5 mice per genotype. **i** Reduced myosin-4 protein expression in C2C12 cells treated with PRSS56. **j** Schematic of HM etiology.

Since PRSS56 is a secreted protein with trypsin-like serine protease activity, we next aimed to explore its potential substrates or downstream effectors to determine how it regulates ocular axial length. We performed quantitative proteomic analysis using global tandem mass tag labeling on paired enucleated whole ocular globes collected from eyes injected with AAV2-PRSS56^WT^ vs AAV2-PRSS56^Mut^ and eyes injected with AAV2-PRSS56^WT^ vs AAV2-eGFP at two months of age. Overall, 31,308 peptides corresponding to 4681 proteins were identified, 4126 of which were quantified (Supplementary information, Fig. S6a, b). Principal component analysis clearly distinguished the six eyeball samples into two distinct categories (Supplementary information, Fig. S6c, d). The comparison between PRSS56^WT^ and the empty vector revealed 6 significantly downregulated proteins and 14 upregulated proteins, and the comparison between PRSS56^WT^ and PRSS56^Mut^ revealed 9 downregulated proteins and 5 upregulated proteins (*P* < 0.05, fold change > 1.5) (Fig. 5a). Notably, the differentially expressed proteins shared by both comparisons included myosin-4 and keratin, type I cytoskeletal 14 (Fig. 5b).

Consistent with the proteomic results, eyes overexpressing PRSS56^WT^ showed reduced myosin-4 signal relative to eyes overexpressing PRSS56^Mut^ in whole-eye immunoblots (Fig. 5c). To determine the tissue origin of this signal, we performed region-resolved analyses. Semi-qPCR detected *Myh4* transcripts in preparations of both the dissected iris-ciliary body region and scleral tissue (Fig. 5d), indicating that *Myh4* mRNA is present in multiple compartments. IF analysis further confirmed the localization of myosin-4 protein in the posterior sclera and the ciliary region. Notably, Myosin-4 IF intensity was consistently reduced in AAV2-PRSS56^WT^ eyes compared with AAV2-PRSS56^Mut^ eyes (Fig. 5e, f; Supplementary information, Fig. S6e).

We next microdissected scleral tissue from DOX-induced Tet-on PRSS56 high-expression mice and WT littermates and performed sclera-specific western blot for myosin-4. These western blot analyses reproduced the differences observed in whole-eye lysates, with reduced Myosin-4 abundance in the sclera of PRSS56-overexpressing animals compared with controls (Fig. 5g, h). As reliable biochemical isolation of the ciliary body is technically challenging, we instead performed in vitro validation assays. The addition of enriched, immunoprecipitated active PRSS56 recombinant protein to differentiated C2C12 myotubes following horse serum induction markedly reduced myosin-4 protein levels in this cellular model (Fig. 5i).

Taken together, the proteomic analysis, sclera-specific western blotting, and in vitro validation indicate that increased PRSS56 expression is associated with reduced myosin-4 abundance in ocular tissues, including the sclera and ciliary region (Fig. 5j).

### Blue light mitigates axial elongation by suppressing *Prss56* upregulation in myopia-model animals

**Fig. 6:**
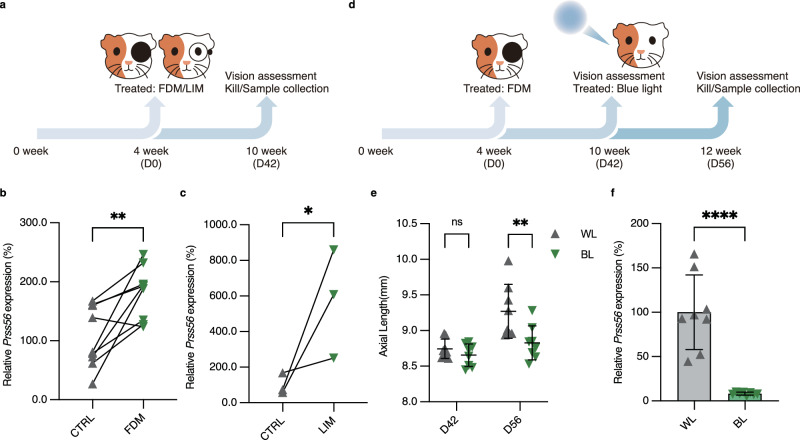
Blue light exposure reduces axial elongation in myopia-modeled animals by suppressing *Prss56* upregulation. **a** Schematic representation of myopia modeling via FDM and LIM methods in guinea pigs. **b**, **c** Increased *Prss56* mRNA levels in the retinas of FDM guinea pigs (**b**) and LIM guinea pigs (**c**) relative to matched controls; *n* = 3–9 guinea pigs per condition. **d** Schematic of the blue-light exposure intervention in FDM-modeled guinea pigs. **e** Blue-light exposure for 2 weeks significantly attenuated axial elongation in FDM guinea pigs compared with white-light controls; *n* = 8 guinea pigs per condition. **f** Reduced *Prss56* mRNA levels in the retinas of FDM-modeled guinea pigs following blue light exposure; *n* = 8 guinea pigs per condition.

Having consistently observed the induction of myopia upon increased *Prss56* expression using various methodologies, we next examined *Prss56* expression levels in non-genetic models of myopia. To this end, we generated two pigmented guinea pig models of HM through form-deprivation myopia (FDM) and lens-induced myopia (LIM) (Fig. 6a). After 42 days of treatment, refractive assessments indicated that both FDM and LIM induced myopic refractive errors in the treated eyes, which exhibited significantly greater myopic shifts, together with increases in axial length, anterior chamber depth, and lens thickness (Supplementary information, Fig. S7a–h). *Prss56* expression levels were significantly upregulated in both the FDM and LIM retinae compared with the contralateral eye in the guinea pig models (Fig. 6b, c). This finding suggests that elevated PRSS56 expression may serve as a potential biomarker of myopia as well as a causative factor. Interestingly, in the FDM guinea pig model, two weeks of blue-light exposure applied after occluder removal significantly reduced the rate of further axial elongation compared with age-matched white-light controls (Fig. 6d, e; Supplementary information, Fig. S8a–d). Critically, this intervention also suppressed retinal *Prss56* expression, suggesting that a lighting condition with increased short-wavelength content was associated with reduced *Prss56* expression in vivo (Fig. 6f). We note, however, that the blue-light and white-light conditions were not matched for total irradiance or for species-specific α-opic activation of photoreceptors (Supplementary information, Table S7). Although our current experiment does not allow separation of wavelength-specific effects from those of total light dose, the observed association of blue-light exposure with reduced *Prss56* expression and axial elongation is consistent with several experimental studies reporting that short-wavelength light, including the blue-violet component of the spectrum, can suppress myopia progression.^28–37^ Human epidemiological studies indicate that greater time spent outdoors is associated with a lower incidence and slower progression of myopia.^38–40^ Outdoor light is characterized by higher overall illuminance and broader spectral irradiance, which includes increased short-wavelength content relative to typical indoor lighting.^41^ Our research may provide a possible molecular basis for this observation, as we demonstrated that increased *Prss56* is a causal factor and biomarker of myopia, as well as a photosensitive target that can be downregulated by blue light to improve visual health. As a key determinant of ocular axial length, increased expression of *PRSS56* is associated with axial elongation and the onset of myopia. Conversely, factors that limit *PRSS56* expression could provide potential strategies to mitigate myopia, particularly by reducing elevated PRSS56 levels in children and adolescents with HM.

## Discussion

We set out to identify causal genetic variations underlying early-onset HM and to define the molecular consequences of such variants. Using two large multigenerational families, sporadic cases, patient-derived iPSCs and organoids, and complementary mouse models, we showed that rare non-coding variants in the *PRSS56* promoter led to increased promoter activity in vitro and an axial myopia phenotype in vivo. These results link a specific regulatory lesion to elevated PRSS56 output and to ocular axial elongation, providing a mechanistic bridge from genetic association to phenotype. We acknowledge that direct confirmation of allele-specific upregulation in patient ocular tissues remains desirable, although surgical specimens are rarely available.

At the population level, large GWAS meta-analyses have highlighted a predominantly polygenic architecture for refractive error, with many risk signals mapping to non-coding sequences.^9,10^ However, statistical association alone does not reveal causality or mechanism. Our study illustrates how targeted, function-oriented follow-up of rare non-coding alleles can move from association to mechanism, a strategy aligned with the growing recognition that promoter variants explain a substantial fraction of rare-disease genetic burden.^42^ The *PRSS56* promoter variant co-segregated with disease, increased reporter activity, and was associated with axial elongation and a myopic refractive shift in an orthogonal KI model and inducible overexpression models. Together, these strands of evidence support the view that *PRSS56* promoter variants constitute independent genetic risk factors for hereditary axial HM and indicate an important role for PRSS56 in controlling ocular axial growth, even in the context of a generally polygenic trait.

Mechanistically, our data support a *cis*-regulatory model in which changes in the promoter sequence alter transcription factor binding and thereby modulate *PRSS56* transcription. EGR1 (ZENK) emerges as a candidate regulator because of the allele-dependent binding observed in our EMSA and reporter assays. In our models, PRSS56 perturbation did not affect EGR1 expression levels (Supplementary information, Fig. S9a–d), suggesting that the identified promoter variants act in *cis* rather than through changes in EGR1 abundance.

Although EGR1 has previously been implicated in ocular growth regulation, its functional effects appear to be context-dependent.^43,44^ Consistent with this complexity, we observed that short-wavelength light exposure reduced *Egr1* expression in BALB/c animals in pilot experiments (Supplementary information, Fig. S9e), indicating that EGR1 does not act as a simple, invariant protective factor across all contexts. Together, our findings indicate that EGR1 contributes to PRSS56 regulation primarily through promoter-specific interactions and that its role in eye growth is modulated by genetic and environmental context.

Our data suggest multiple possible downstream pathways by which elevated PRSS56 could influence ocular growth. IF revealed that PRSS56 was localized to inner retinal layers and regions adjacent to the RPE, a distribution compatible with secretion toward both the vitreal and subretinal sides of the retina. Representative staining suggested reduced myosin-4 signal adjacent to the ciliary region in AAV2-PRSS56^WT^ eyes. Although alterations in the ciliary muscle have been implicated in accommodation-related myopia in humans,^45^ the murine ciliary muscle is anatomically rudimentary, shows limited contractile function, and has no well-established link to myopia in mice. Consequently, these data were interpreted as correlative rather than as evidence for a ciliary-body-driven mechanism. By contrast, several independent lines of evidence pointed to involvement of the sclera. *Myh4* transcript abundance was high in dissected sclera, and sclera-specific western blot showed reduced myosin-4 protein in PRSS56-overexpressing animals. Taken together, these findings are most consistent with a model in which PRSS56 influences scleral molecular composition and in which such scleral remodeling contributes to axial elongation. The possibility of ciliary-body involvement was not excluded, but it remains unproven in the absence of quantitative morphometry and tissue-specific perturbation experiments.

We complemented our genetic and anatomical studies with environmentally based experiments motivated by initial observations that ambient light modulates *Prss56* expression in KI animals (Supplementary information, Fig. S9f). In guinea pig cohorts modeling pathological myopia, short-wave (blue) light exposure applied after occluder removal was associated with attenuation of subsequent axial elongation and with reduced retinal *Prss56* levels. As the illumination experiments were performed after removal of form deprivation and, under our experimental conditions, axial elongation continued rather than undergoing rapid spontaneous recovery,^46^ blue-light exposure did not reverse established axial elongation but significantly attenuated further axial progression relative to that in contemporaneous white-light controls. We therefore interpret the post-form deprivation illumination data as hypothesis-generating evidence that light modulates *Prss56* expression and can slow subsequent pathological progression in a model approximating pathological myopia, but not as definitive proof of FDM rescue.

In addition, because the blue-light condition increased overall photoreceptor activation as well as short-wavelength content, the present design did not allow the effects of spectral composition to be disentangled from those of total light dose. Given this confounding of spectrum and dose, conclusions about wavelength specificity require caution. Although violet or near-ultraviolet wavelengths may exert related biological effects because of overlapping action spectra, species differences in photoreceptor sensitivity and ocular media transmission, as well as safety considerations, make blue light a more immediately translatable focus.^31,35^ Careful matching by biologically relevant irradiance metrics and head-to-head wavelength comparisons will be required before clinical translation is considered.

Complementary to an environmental approach, molecular therapies targeting PRSS56 offer a direct way to interrupt the putative pathogenic pathway. Previous work has proposed targeting downstream mediators of PRSS56 to counteract excessive eye growth,^47^ and our findings extend this rationale by showing that both increased PRSS56 expression and increased PRSS56 protease activity contribute to axial elongation, thus supporting two complementary strategies. One strategy is to reduce PRSS56 expression using gene expression modulators such as RNA interference, antisense oligonucleotides, or CRISPR interference delivered in a tissue-selective manner. An alternative strategy is to inhibit PRSS56 enzymatic function using small molecules. We preliminarily identified several small molecules that reduce PRSS56 activity in vitro, including a compound denoted TLCK (tosyl-L-lysyl-chloromethane hydrochloride), and these molecules provide a starting point for medicinal chemistry optimization and specificity testing. Translation of small-molecule or oligonucleotide approaches will require demonstration of target engagement and efficacy in organoids and animal models, followed by systematic pharmacokinetic and safety evaluation.

Gene-based interventions represent a promising third avenue for therapeutic development, particularly strategies aimed at reducing PRSS56 expression or activity. Potential approaches include vector-based delivery of gene silencers, allele-specific suppression where appropriate, and precision genome editing aimed at normalizing promoter activity. Each of these approaches raises distinct challenges, including choice of delivery route, cell-type specificity, durability of effect, and potential off-target consequences. Emerging delivery platforms, such as nanomaterial-based carriers that enable targeted retinal delivery, may help to address some of these challenges by improving tissue specificity and reducing systemic exposure.

In conclusion, our findings support the involvement of non-coding functional variants in the *PRSS56* promoter as a significant causal factor in the development of HM. The positive correlations of these variants with increased PRSS56 expression and ocular elongation provide a clearer understanding of how the genetic landscape influences myopia. Future studies should focus on the racial diversity of rare *PRSS56* promoter variants in broader populations and on environmental factors, including external light and internal metabolites, that may modulate PRSS56 expression. This research contributes to our understanding of the genetic basis of myopia, paving the way for potential therapeutic strategies targeting PRSS56 for the management of refractive errors in large and widespread groups of children and adolescents with myopia. The pathway from genetic predisposition to myopia underscores the importance of integrated approaches that combine genetics, molecular biology, and clinical insights to tackle complex ocular disorders.

## Materials and methods

### Subjects

Two multigenerational HM families were identified from records of the EYE & ENT Hospital of Fudan University, Shanghai, China. No consistent extraocular or systemic abnormalities were identified; ocular comorbidities such as cataracts and lens dislocation were observed in a subset of affected individuals. Peripheral blood samples were collected for the extraction of genomic DNA. For each family member, refractive error, intraocular pressure, and axial length were measured using standard ophthalmic examinations. An additional 236 sporadic patients with eoHM and 653 matched normal controls were recruited for mutation screening by Sanger sequencing. Written informed consent was obtained from all participants or their legal guardians prior to the study. The study conformed to the tenets of the Declaration of Helsinki and was approved by the Committee for Ethics and Research at the EYE & ENT Hospital, Fudan University (Approval No. 20170301-YZQ).

### Cell culture

MIO-M1, HEK293T, and C2C12 cells were cultured in Dulbecco’s Modified Eagle’s Medium (DMEM; Gibco, NY, USA), and RPE1 cells in DMEM/F12 (Gibco), both supplemented with 1% penicillin/streptomycin (penicillin 100 U/mL and streptomycin 10 μg/mL; Gibco) and 10% fetal bovine serum (Gibco), and preserved using CELLSAVING (New Cell & Molecular Biotech, Suzhou, China). Human iPSCs were maintained in mTeSR 1 Complete Kit medium (STEMCELL, Canada) containing the same concentrations of antibiotics.

### Whole-genome genotyping and linkage analysis

Analyses were performed according to the manufacturer’s protocol for the Illumina Human OmniZhongHua-8 BeadChip (> 890,000 SNPs) using DNA samples from 21 members of Family 1. Parametric linkage analysis, assuming a 95% penetrant, autosomal-dominant model with a disease allele frequency of 0.01, was performed using MERLIN (v1.12) as described previously.^48^

### CNV analysis

CNVs were identified using aCGH. Genomic DNA from 1 affected (Ⅲ-8) and 1 unaffected individual (Ⅲ-3) in F1 was hybridized to the Agilent SurePrint G3 1 × 1 M array (median probe spacing of 1 per 2.1 kbp). Human male genomic DNA (P/NG1471; Promega Corporation, WI, USA) was used as a reference for labeling. Data were extracted using Agilent Feature Extraction software (v10.7.3.1) and analyzed with Genomic Work bench (v7.0.4) (Agilent Technologies). Subsequently, a statistical sensitivity threshold of 6.0 was set for the Aberration Detection Method 2 algorithm. The threshold settings for a positive call were 6.0 for sensitivity and 3 for the minimum number of probes per region. No pathogenic germline CNVs associated with eye development were detected in this study. Detailed information is provided in Supplementary information, Table S2.

### Whole-exome sequencing and WGS

Whole-exome sequencing was performed using the Agilent SureSelect XT2 Human All Exon v.4.0 capture kit and the Illumina HiSeq 2000 sequencer for six members of F1, including four affected (Ⅱ-2, Ⅲ-8, Ⅳ-1, Ⅳ-3) and two unaffected (Ⅲ-3, Ⅲ-6) individuals. Reads were aligned to the human reference sequence (UCSC Genome Browser hg19) using the Burrows–Wheeler Alignment tool, and duplicate reads were removed. Single-nucleotide variants and small InDels were called using the Genome Analysis Toolkit (GATK v1.4) in parallel with SAMtools (v0.1.19) and were annotated with ANNOVAR. ExomeDepth was used to identify CNVs. Functional predictions were assessed using PolyPhen-2 and MutationTaster. Detailed information is provided in Supplementary information, Table S3.

WGS was performed using the TruSeq Nano DNA library preparation kit and the Illumina HiSeq X platform for two affected individuals (Ⅱ-2, Ⅲ-1) from F1. Reads were processed according to the aforementioned procedures. Public databases, including dbSNP137, the 1000 Genomes Project, and the HapMap 8 database, were used for subsequent genetic analysis. Detailed information is provided in Supplementary information, Table S4.

### *PRSS56* sequencing

The segregation of c.-187G>T in F1 and c.-187G>C in F2, identified by WGS, was confirmed by Sanger sequencing. PCR was performed using Taq HotStart DNA Polymerase (TaKaRa Bio, Shiga, Japan). Products were purified using Exon/SAP and sequenced using the BigDye Cycle Sequencing Kit (Thermo Fisher Scientific, MA, USA) on an ABI 3730 Genetic Analyzer (Applied Biosystems, CA, USA). A region 603 bp upstream of the translation initiation site in *PRSS56* was screened for variants in 236 sporadic cases of eoHM and 653 matched normal cṭoṭntrols. Details of the genotyping results and sequencing primers are provided in Supplementary information, Tables S5 and S6.

### Plasmid construction

Human EGR1 was PCR-amplified from the pCMV3-EGR1-HIS plasmid purchased from Sino Biological (Beijing, China) and inserted into the pCMV6-AC-HA empty vector using the AsiSI–MluI restriction sites. A 603-bp fragment of the human *PRSS56* promoter (positions −709 to −107) was PCR-amplified from genomic DNA and subcloned into the pGL3-Basic vector using the *NheI*–*HindIII* restriction sites. Each of the mutations identified in the *PRSS56* promoter was generated by site-directed mutagenesis using the Mut Express MultiS Fast Mutagenesis Kit (Vazyme, Nanjing, China). The ORF of mouse PRSS56 was PCR-amplified from cDNA and inserted into the pCMV6-Entry vector at the AsiSI–MluI sites. The primers used for plasmid construction are shown in Supplementary information, Table S6. All recombinant plasmids were confirmed by DNA sequencing.

### Luciferase assays

MIO-M1, RPE1, and HEK293T cells were seeded in 24-well culture plates and co-transfected with 250 ng pCMV6-EGR1-HA, 250 ng *PRSS56*-promoter^WT^/*PRSS56*-promoter^Mut^/empty vector plasmid, and 10 ng Renilla luciferase plasmid (pRL-CMV) for normalization using Lipofectamine 3000 (Invitrogen, MA, USA). At 36 h after transfection, cells were lysed and assayed for Renilla and firefly luciferase activity using a dual-luciferase reporter assay system (Promega, WI, USA) according to standard procedures. At least four independent repeats were performed.

### EMSA

HEK293T cells seeded in 10-cm culture plates were transfected with 10 μg pCMV6-EGR1-HA using Lipofectamine 3000 (Invitrogen). At 48 h after transfection, nuclear protein was prepared using NE-PER Nuclear and Cytoplasmic Extraction Reagents (Thermo Fisher Scientific). A double-stranded DNA probe containing *PRSS56* c.-187G/T/C was generated by annealing the oligonucleotide and its complementary oligo. The oligonucleotides were labeled with biotin at the 5′ end or were unlabeled. The protein–DNA binding assay was performed according to the instructions of the LightShift Chemiluminescent EMSA Kit (Thermo Fisher Scientific). In supershift experiments, nuclear extracts were incubated with 1 μL anti-EGR1 antibody (Cell Signaling Technology, MA, USA) for 10 min at room temperature. All samples were resolved by electrophoresis on 6.5% polyacrylamide gels (Beyotime, Shanghai, China). Oligonucleotides are provided in Supplementary information, Table S6.

### Establishment of iPSCs

Fresh peripheral blood samples (10 mL) from an affected individual (F1-Ⅲ-8) and her sibling (F1-Ⅲ-7) were collected for iPSC reprogramming and generation by IxCell Co., Ltd. (Shanghai, China). In brief, four transcription factors (OCT3/4, SOX2, c-Myc, and Krüppel-like factor 4) were transfected into human peripheral blood mononuclear cells via electroporation. The resulting iPSC clones were screened by standard identification procedures, including karyotyping, immunostaining, and flow cytometry for the stem cell markers NANOG, OCT4, and SOX2; evaluation of mesoderm, ectoderm, and endoderm differentiation; and detection of exogenous transcription factors. The associated images are provided in Supplementary information, Fig. S3a, b.

### Differentiation of retinal organoids

Retinal organoids were generated from two iPSC lines according to a published method.^49^ In brief, from day 1 to day 7 (D1–D7), iPSCs were cultured in suspension to form early neural aggregates. From day 8 to day 15 (D8–D15), the aggregates were transferred to adherent conditions to promote proliferation. Between day 7 and day 27 (D7–D27), cells gradually differentiated into retinal-like structures. On day 28 (D28), the cultures were returned to suspension conditions to facilitate the development of retinal organoids and the emergence of RPE cells. After day 42 (D42), treatment with retinoic acid and taurine further promoted the maturation of structurally organized retinal organoids.

### *Prss56* KI, KO, and transgenic (PRSS56-Tet-on) mice

For animal studies, all experimental protocols were performed in accordance with institutional ethical guidelines and approved by the Animal Care and Ethics Committee of the Laboratory Animal Center, Fudan University (2023-FCYY-123JZS). The production of Cas9 mRNA and sgRNA, as well as the generation of KI mice, has been described previously.^50^ In brief, C57BL/6 J female mice (7–8 weeks old) were used as embryo donors. Superovulation was induced by intraperitoneal injection of pregnant mare serum gonadotropin and human chorionic gonadotropin, followed by mating with C57BL/6J male mice. Fertilized embryos (zygotes) were collected from the oviducts, and Cas9 mRNA (100 ng/μL) and donor vector (50 ng/μL) were mixed and injected into the cytoplasm of fertilized eggs with both pronuclei visible in Chatot–Ziomek–Bavister medium. The injected zygotes were then cultured in Quinn’s Advantage cleavage medium (In Vitro Fertilization, Inc.) containing SCR7 (50 μM; TOCRIS Bioscience, Bristol, UK) for ~24 h, and 18–20 two-cell-stage embryos were transferred into the oviducts of pseudo-pregnant ICR female mice at 0.5 d *post coitum*. Genomic DNA was extracted from tail tips and genotyped by sequencing. Sequences of sgRNAs are provided in Supplementary information, Table S6.

For transgenic (Prss56-Tet-on) mice, the constructed targeting plasmid (20 ng/μL), sgRNA (2 ng/μL), and Cas9 mRNA (5 ng/μL) were mixed and microinjected into NOD-SCID fertilized eggs. The injected fertilized eggs were then transferred into the oviducts of pseudo-pregnant ICR female mice to enable pregnancy and the birth of F0-generation pups. Genomic DNA was extracted from F0-generation mice born to the recipient mice, and PCR and sequencing were performed to confirm their genotypes. After the positive F0-generation mice reached sexual maturity, they were bred with WT-background mice, and genomic DNA was extracted from the resulting F1-generation pups for PCR and sequencing analysis.

All mice used in this study were genetically confirmed to be free of the *Crb1-rd8* mutation.

### DOX-induced overexpression of PRSS56

Transgenic mice carrying both the tetracycline transactivator and the TetO responder cassette were induced to overexpress PRSS56 by administration of DOX in the drinking water. Doxycycline hyclate was prepared at 2 mg/mL in 5% (w/v) sucrose and supplied from three weeks of age until phenotypic analysis at six weeks. Drinking water was supplied in amber bottles and replaced every three days. Control animals were WT littermates that received identical DOX-containing drinking water for the same period to control for any off-target effects of DOX. Water consumption was monitored periodically, and no overt adverse effects related to DOX administration were observed.

### Virus generation and intravitreal infection

AAV2-CMV-eGFP (10^10^ vector genomes/μL; S0263-2) was purchased from Shanghai Taitool Bioscience. AAV vectors were packaged into the AAV2 serotype as described previously.^51^ AAV vectors and helper plasmids were co-transfected into HEK293T cells. Cells were harvested 96 h after transfection, and viral particles were released from the cells by freeze-thaw cycles and sonication. Virus was purified using cesium chloride density-gradient ultracentrifugation and dialyzed in HEPES buffer (20 mM HEPES and 145 mM sodium chloride, pH 7.8). Viral titers were measured by qPCR and were typically 10^10^ vector genomes/μL. Mice were infected by intravitreal injection as described previously.^52^ Glass micropipettes (tip outer diameter ≈ 30–50 μm) were prepared using a puller and connected to a Hamilton syringe. Day-old mouse pups (P1) were anesthetized by chilling on ice; the eye was then exposed by making a small incision in the eyelid near the lens with ophthalmic scissors. Using a micromanipulator and a Hamilton microinjector, 0.5 μL of viral suspension was delivered slowly into the vitreous cavity through a trans-scleral approach 0.5–1 mm posterior to the limbus, taking care to avoid the lens. The pipette was maintained in place for ~20–30 s after injection to minimize reflux. Animals were returned to a 38 °C warming pad for 5–10 min and then returned to the mothers for nursing. All procedures conformed to institutional animal care and use guidelines.

### FDM and LIM guinea pig models

FDM and LIM models were established in 4-week-old healthy pigmented guinea pigs using established protocols with custom optimizations, as detailed below.^33,53^

For FDM, under brief light isoflurane anesthesia, the right eye of each animal was fully occluded with a custom-made opaque eye patch affixed to the periocular skin using a skin-safe tissue adhesive and secured with a soft elastic headband to minimize displacement while avoiding pressure on the globe. The contralateral (left) eye served as an untreated internal control. Occlusion was maintained continuously for six weeks, and patch placement and integrity were verified twice daily throughout the treatment period. Animals were inspected daily for general health and ocular signs (blepharospasm, discharge, conjunctival hyperemia, corneal opacity). The occluder design restricted direct paw-to-eye contact, and transient irritation was managed with topical ocular lubricant; any animal with persistent ocular pathology or signs suggestive of infection would have been removed from the study and excluded from analysis.

For LIM, a custom-fabricated 20-mm −10-diopter resin lens was mounted ~2–3 mm anterior to the right cornea using a lightweight plastic frame affixed to the periocular skin with medical-grade adhesive. This design ensured that the lens did not directly contact the cornea, allowing adequate airflow and minimizing the risk of irritation. The contralateral eye served as the internal control. Lenses were worn continuously for six weeks, with attachment and cleanliness checked twice daily. If a lens became dislodged or contaminated, it was promptly cleaned or replaced under brief anesthesia. Animals were examined daily for ocular surface integrity and general health; no sustained lens-related irritation or infection was observed.

All procedures followed established small-animal myopia protocols and were performed in accordance with institutional animal care and use committee approvals.

### Blue-light interventions

At six weeks after induction of form deprivation, the occluders were removed, and the guinea pigs were returned to unrestricted binocular vision. Animals were randomized into a blue-light treatment group and a white-light control group, ensuring comparable distributions of sex, weight, and baseline refractive parameters. During the subsequent 2-week intervention period, the blue-light cohort was exposed to LED illumination centered at 440 nm (Ruiliu Electronic Co., Dongguan, China), whereas the control group was exposed to a standard white LED light (color temperature 5000 K). Spectral power distributions (SPDs) of the blue and white sources were measured at the animal eye position with a calibrated spectroradiometer (HPCS-330, Hopoocolor, Hangzhou, China). Photoreceptor-weighted irradiances were calculated using guinea pig-specific spectral sensitivity functions in the Alpha-Opics Animal Toolbox (https://alphaopics.shinyapps.io/animal_light_toolbox/), which incorporates species-appropriate photoreceptor action spectra and ocular media transmission.^54,55^ Guinea pig photoreceptor-weighted irradiances for each illumination condition are provided in Supplementary information, Table S7. SPDs and x,y chromaticities plotted in the CIE 1931 color space are provided in Supplementary information, Fig. S8a–d. Animals were housed in environmentally controlled chambers under a standard 12-h light/dark cycle for 2 weeks, as described previously.^33,34^

### RT-qPCR

Retinas were carefully dissected from mice and guinea pigs under RNase-free conditions. Animals were euthanized following institutional ethical guidelines, and eyes were immediately enucleated and immersed in ice-cold RNase-free PBS. Under a stereomicroscope, the cornea and lens were removed, and the neural retina was gently separated from the RPE and choroid using fine forceps. The isolated retinas were briefly rinsed in PBS, blotted dry on sterile filter paper, snap-frozen in liquid nitrogen, and stored at −80 °C until RNA extraction.

Total RNA was extracted from the isolated retinal tissues, as well as from iPSCs and retinal organoids, using VeZol Reagent (Vazyme), and first-strand cDNA was synthesized using HiScript III Reverse Transcriptase (Vazyme). RT-qPCR was performed using ChamQ Universal SYBR qPCR Master Mix (Vazyme) on an ABI StepOnePlus instrument. The ΔΔCt algorithm was used to quantify changes in gene expression, using the housekeeping gene *GAPDH/Gapdh* for normalization. Primer sequences are provided in Supplementary information, Table S6.

### Ocular biometrics

All ocular measurements were performed under anesthesia, with animals maintained on a heating pad to preserve body temperature.

Animals were anesthetized with isoflurane delivered via a precision vaporizer: induction with 3%–4% isoflurane in oxygen and maintenance at 1.5%–2% during measurements. Adequate depth of anesthesia was confirmed by the absence of the pedal withdrawal reflex. Cycloplegia was induced by instillation of one drop of 0.5% tropicamide phenylephrine (Santen) in each eye after anesthesia.

Refraction was measured in darkness along the vertical pupil meridian using an eccentric infrared photorefractor designed by Schaeffel.^56^ Animals were positioned and aligned until a clear first Purkinje image appeared at the center of the pupil, indicating an on-axis measurement. At least three independent readings were acquired for each eye, and the mean value was used for analysis.

Corneal curvature was assessed using a custom SD-OCT system.^57,58^ Images of the anterior corneal surface were acquired and analyzed to determine the corneal radius of curvature from the anterior corneal contour. Three repeated OCT measurements were obtained for each eye, and the mean value was used for analysis.

Axial length was measured by A-scan ultrasonography in KI mice, Tet-on mice, and guinea pigs using a high-frequency ophthalmic A-scan ultrasound biometer appropriate for the eyes of small animals (KN-1800, Kangning, Wuxi, China). The instrument was operated in the manufacturer’s ocular mode and calibrated with an ocular phantom according to the manufacturer’s instructions before data collection. Measurements were obtained using a contact technique, with the probe aligned perpendicular to the central cornea. Multiple A-scan traces were recorded per eye, and on-axis traces without evidence of corneal compression or poor signal were retained. The mean of at least five consistent on-axis measurements for each eye was used for analysis; scans were repeated if the standard deviation among accepted measurements exceeded pre-defined acceptance criteria.

Axial length was quantified in AAV-injected mice by custom SD-OCT. Anesthetized mice were placed in a cylindrical holder mounted on a motorized positioning stage in front of a modified slit-lamp assembly. The eye was aligned along the optical axis using an X-Y cross-scanning system to ensure on-axis imaging. SD-OCT scans along the visual axis were acquired, and raw data were exported for post-processing with custom software to calculate axial length. Three independent scans were obtained for each eye, and the mean value was used for statistical analysis.

For all modalities, on-axis alignment and signal quality were confirmed visually. Obvious outliers, traces showing corneal compression, or scans with poor signal were excluded. All raw data and representative traces/images were saved for verification.

After completion of the measurements, animals were allowed to recover in a warm chamber until fully awake and were then returned to their home cages.

### Proteomics

Tandem mass tag-based proteomic analysis was supported by Jingjie PTM Biolabs (Hangzhou, China) as described. Protein identification was performed with a confidence threshold of 95%, and only proteins with *P* < 0.05 were considered for quantification and further validation. For visualization and exploratory analysis, normalized protein abundance values were used for principal component analysis (PCA) and hierarchical clustering. The resulting PCA plots and heatmaps were used to assess the overall consistency and grouping of biological replicates.

### Immunoprecipitation

HEK293T cells seeded in 10-cm culture plates were transfected with 10 μg pCMV6-PRSS56-Flag using Lipofectamine 3000 (Invitrogen). At 48 h after transfection, cells were lysed in non-denaturing buffer (C510013, Sangon Biotech), and anti-Flag immunoprecipitation was performed using Pierce Anti-DYKDDDDK Magnetic Agarose (Thermo Fisher Scientific) following standard procedures. 3× Flag peptide (Beyotime) was used to elute active PRSS56 recombinant protein from the beads.

### H&E staining and IF

Eyes were enucleated and fixed in 4% PFA for 4 h at room temperature before preparation of paraffin sections. H&E and IF staining were performed on sections prepared from paraffin-embedded tissues according to standard protocols. After blocking with 10% normal goat serum, slides were incubated with primary antibodies against Flag (14793, Cell Signaling Technology, 1:500 dilution), myosin-4 (14-6503-82, Thermo Fisher Scientific, 1:1000 dilution), ZO-1 (21773-1-AP, Proteintech, 1:1000 dilution), and ACTA2 (14395-1-AP, Proteintech, 1:1000 dilution) overnight at 4 °C. Slides were then incubated with secondary antibodies (goat anti-rabbit or anti-mouse IgG, Alexa Fluor 488 or 594; Thermo Fisher Scientific, 1:1000 dilution) for 2 h, followed by nuclear counterstaining with DAPI (Thermo Fisher Scientific) in PBS for 6 min. Confocal imaging was performed using an SP8 system (Leica), and images were processed using the Leica AF software suite.

### Transmission electron microscopy

The sclera was cut into ~2-mm^3^ pieces and fixed in 2.5% glutaraldehyde (Excellence in Brain Science and Intelligence Technology, Chinese Academy of Sciences). The tissues were processed as described previously^59^ and observed using a JEM-1230 electron microscope (JEOL Ltd., Japan).

### Immunoblotting

For immunoblot analysis, protein lysates were prepared from cultured cells, retinal organoids, and ocular tissues.

Cells were washed twice with ice-cold PBS and lysed directly in RIPA buffer (P0038, Beyotime) supplemented with protease and phosphatase inhibitors (P1005, Beyotime). Lysates were incubated on ice for 30 min and centrifuged at 12,000 rpm for 10 min at 4 °C. The clarified supernatant was collected for further analysis.

Retinal organoids were collected, washed with ice-cold PBS, and homogenized in RIPA lysis buffer containing protease and phosphatase inhibitors. Samples were incubated on ice for 30 min and briefly sonicated to ensure complete lysis. After centrifugation at 12,000 rpm for 15 min at 4 °C, the supernatant was collected as the total protein extract.

Whole eyes (with optic nerves carefully removed and adherent extra-ocular muscles and connective tissue carefully excised) or microdissected ocular components were freshly collected on ice and homogenized in ice-cold RIPA buffer with protease and phosphatase inhibitors. Homogenization was performed using a handheld tissue homogenizer (OSE-Y50, Tiangen, Beijing, China), followed by sonication. Lysates were incubated for 30 min on ice and centrifuged at 12,000 rpm for 15 min at 4 °C. The supernatant was collected as the total protein.

Protein concentrations were determined using a BCA Protein Assay Kit (23225, Thermo Fisher Scientific).

After electrophoresis and membrane transfer, protein extracts were probed with one of the following primary antibodies in 5% nonfat milk or 5% bovine serum albumin: anti-myosin-4 (14-6503-82, Thermo Fisher Scientific, 1:1000 dilution), anti-PRSS56 (M016393, Abmart, 1:1000 dilution), anti-Flag (14793, Cell Signaling Technology, 1:1000 dilution), anti-COL1 (67288-1-Ig, Proteintech, 1:1000 dilution), anti-ZO-1 (21773-1-AP, Proteintech, 1:1000 dilution), anti-GFP (2956, Cell Signaling Technology, 1:1000 dilution), anti-GAPDH (60004-1-Ig, Proteintech, 1:2000 dilution), or anti-β-tubulin (ab108342, Abcam, 1:1000 dilution).

### Statistics

All data are presented as individual samples and means ± SD. An unpaired two-tailed Student’s *t*-test was used to determine the significance between two groups of normally distributed data using GraphPad Prism software v10.2.1 (GraphPad Software, Inc., CA, USA). Differences were considered significant at *P < 0.05; **P < 0.01; ***P < 0.001; ****P < 0.0001. Comparisons yielding *P* ≥ 0.05 are considered not significant and are labeled as ns in figures.

## Supplementary information

Supplementary Information, Fig. S1

Supplementary Information, Fig. S2

Supplementary Information, Fig. S3

Supplementary Information, Fig. S4

Supplementary Information, Fig. S5

Supplementary Information, Fig. S6

Supplementary Information, Fig. S7

Supplementary Information, Fig. S8

Supplementary Information, Fig. S9

Supplementary Information, Table S1

Supplementary Information, Table S2

Supplementary Information, Table S3

Supplementary Information, Table S4

Supplementary Information, Table S5

Supplementary Information, Table S6

Supplementary Information, Table S7

## Data Availability

The raw WGS sequencing data reported in this study have been deposited at the Genome Sequence Archive in National Genomics Data Center, China National Center for Bioinformation (GSA-Human: subHRA026178) that are publicly accessible at https://ngdc.cncb.ac.cn/gsa-human. Other data supporting the findings of this study are available from the corresponding authors upon reasonable request.
