# Supplementary material for: Increased PRSS56 expression is a causal factor and therapeutic target for human axial high myopia
Source: Cell Res. 2026 Apr 1;36(8):567–81. doi: 10.1038/s41422-026-01241-9 (PMC13424129; doi:10.1038/s41422-026-01241-9)
Supplement: Supplementary file 1 — Supplementary Information, Fig. S1 [file 41422_2026_1241_MOESM1_ESM.pdf]

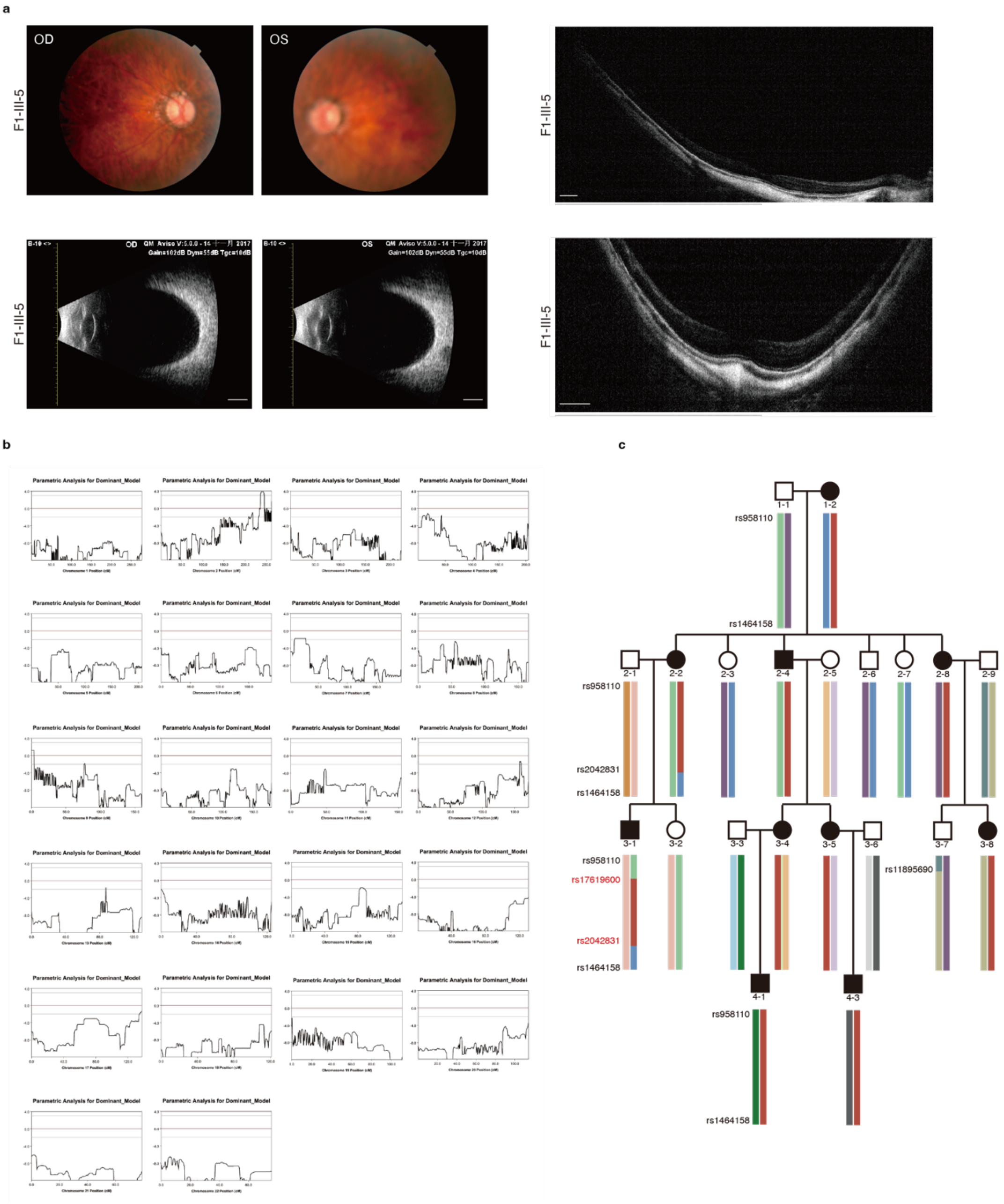


**Supplementary information, Fig. S1 Clinical characterization and locus mapping of HM**

**a** Optical examination on affected individual (F1-III-5) showed leopard-like fundus, temporal papillae of optic papilla and macular degeneration (upper left panel). B-ultrasonic diagnosis showed extreme lengthening of ocular axial length and posterior staphyloma (lower left panel). Scale bar, 5 mm. Optical coherence tomography

indicated the retinal thickness at the fovea decreased (right panel). Scale bar, 250 μm. **b** Genome-wide linkage analysis on 21 individuals of F1 identified a maximum LOD score of 3.81 at rs12621510 in chromosomal region 2q37.1. No other intervals in the remaining 21 autosome pairs yielded LOD scores greater than 1.20. **c** Alleles of SNPs in the region between rs958110 and rs1464158 on chromosome 2q37.1 are arranging for haplotype analysis. Individuals 2-2 and 3-1 carry recombinant chromosomes, suggesting that the HM locus maps between rs17619600 and rs2042831.
