# Supplementary material for: Increased PRSS56 expression is a causal factor and therapeutic target for human axial high myopia
Source: Cell Res. 2026 Apr 1;36(8):567–81. doi: 10.1038/s41422-026-01241-9 (PMC13424129; doi:10.1038/s41422-026-01241-9)
Supplement: Supplementary file 2 — Supplementary Information, Fig. S2 [file 41422_2026_1241_MOESM2_ESM.pdf]

a

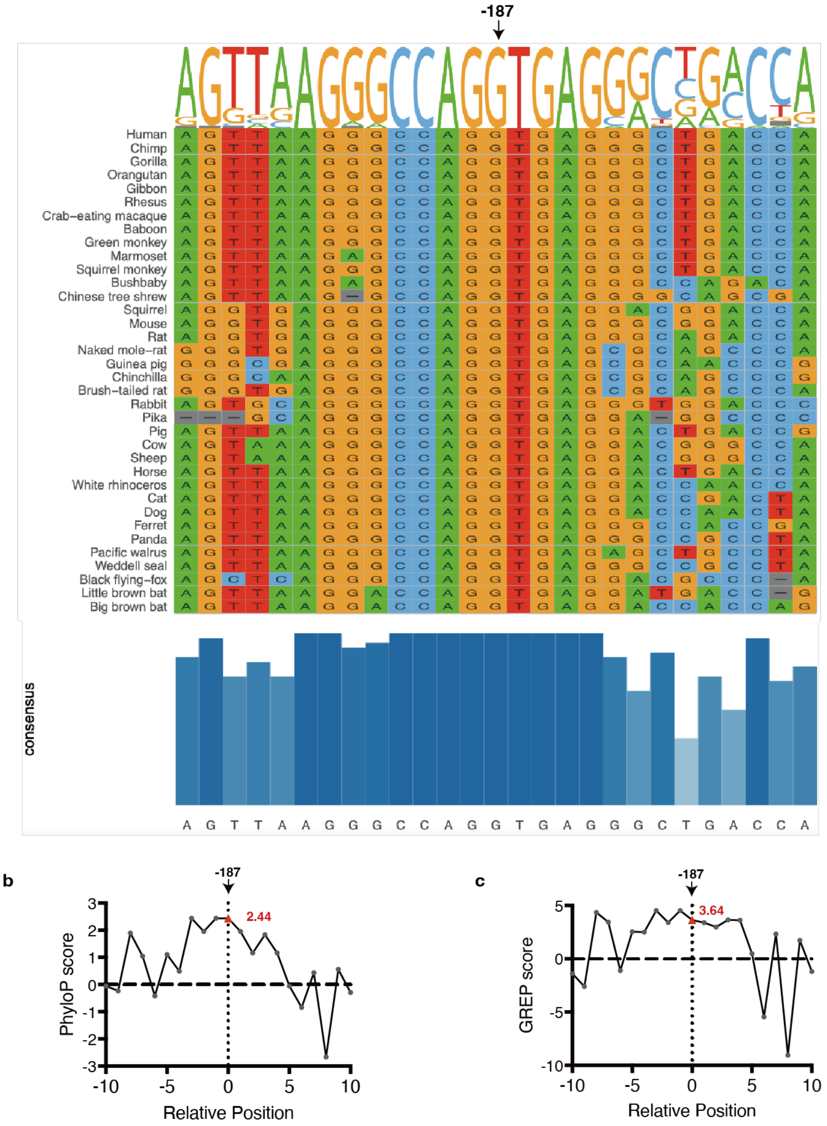

Supplementary information, Fig. S2 Evolutionary conservation of *PRSS56* c.-187

a Conservation of *PRSS56* c.-187 G (black arrow) across 36 mammalian species that possess advanced vision. **b** PhyloP scores at *PRSS56* c.-187 (black arrow) and flanking ±10 bp regions across 46 mammalian species, demonstrating deep evolutionary conservation consistent with functional importance. **c** GERP++ scores at *PRSS56* c.-187 (black arrow) and adjacent ±10 bp regions, revealing reduced mutation frequency compared to neutral expectation.
