# Supplementary material for: Increased PRSS56 expression is a causal factor and therapeutic target for human axial high myopia
Source: Cell Res. 2026 Apr 1;36(8):567–81. doi: 10.1038/s41422-026-01241-9 (PMC13424129; doi:10.1038/s41422-026-01241-9)
Supplement: Supplementary file 3 — Supplementary Information, Fig. S3 [file 41422_2026_1241_MOESM3_ESM.pdf]

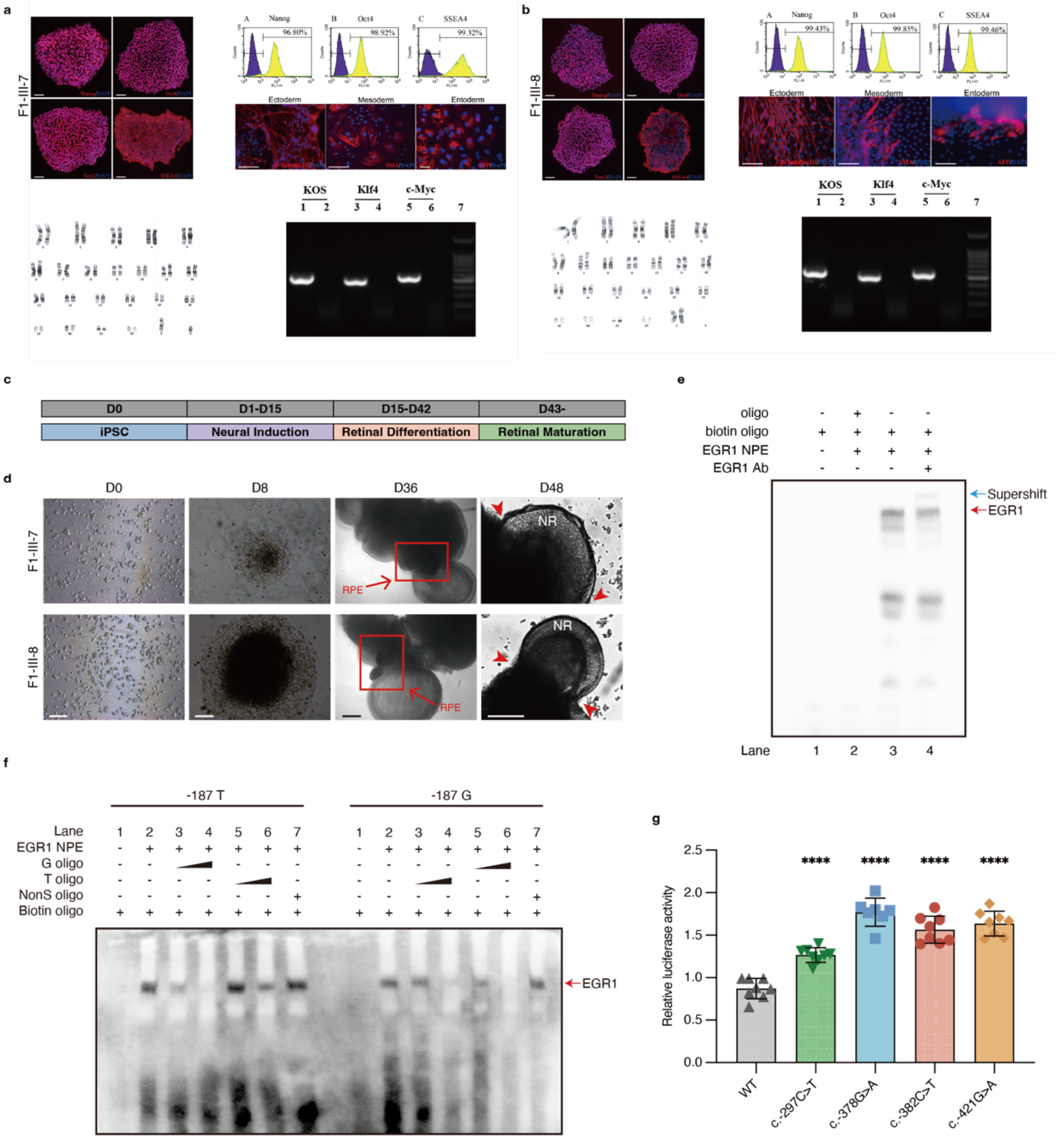

Supplementary information, Fig. S3 Functional validation of *PRSS56* promoter variants

a, b The iPSC clones, F1-III-7 (**a**) and F1-III-8 (**b**), were screened by standard identification procedures, including karyotyping, immunostaining, and flow cytometry for the stem cell markers NANOG, OCT4, SSEA4 and SOX2; evaluation of mesoderm, ectoderm, and endoderm differentiation; and detection of exogenous transcription factors. The representative images are provided. Scale bar, 50 μm. **c** Schematic overview of the stepwise protocol for retinal organoid generation. **d** Representative

bright-field images at key differentiation time points. D0: Undifferentiated iPSCs in suspension. D8: Neural aggregates adhering and proliferating as neurospheres. D36: Optic-cup-like organoid exhibiting a pigmented RPE layer (red box). D48: High-magnification view of a representative retinal organoid showing a continuous neural retina adjacent to RPE (red arrow). Scale bar, 500 μm. **e** Antibody supershift assay confirming the identity of the EGR1-DNA complex. Nuclear extracts prepared from cells transiently overexpressing EGR1 were used in the assay. Addition of anti-EGR1 antibody produced a reproducible supershift (higher-molecular-weight band, blue arrow) and concomitant reduction of the original shifted complex (red arrow). **f** EMSA competition assays demonstrating sequence-specific binding using nuclear extracts from EGR1-overexpressing cells. Graded introduction of unlabeled specific competitor progressively reduced the shifted complex intensity, while scrambled competitor failed to alter the specific complex. The band indicated by the red arrow represents the specific complex formed by EGR1 binding to the biotinylated *PRSS56* promoter probe. **g** Luciferase assays demonstrated that promoter variants identified in sporadic cases significantly enhanced *PRSS56* transcriptional activity; $n = 8$ biological replicates for condition.
