# Supplementary material for: Increased PRSS56 expression is a causal factor and therapeutic target for human axial high myopia
Source: Cell Res. 2026 Apr 1;36(8):567–81. doi: 10.1038/s41422-026-01241-9 (PMC13424129; doi:10.1038/s41422-026-01241-9)
Supplement: Supplementary file 4 — Supplementary Information, Fig. S4 [file 41422_2026_1241_MOESM4_ESM.pdf]

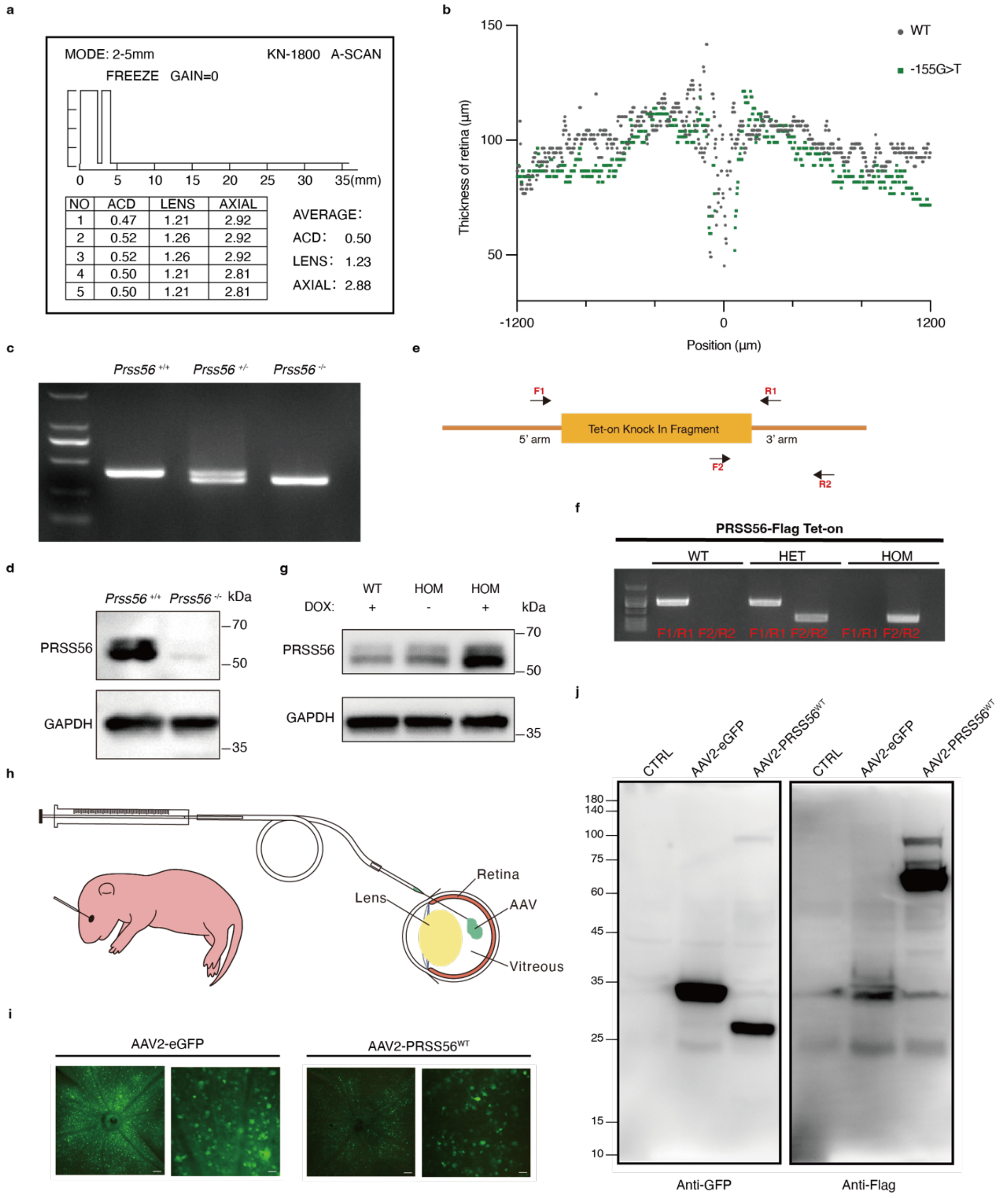

Supplementary information, Fig. S4 Generation and validation of *Prss56* mouse models

a Schematic illustration of A-scan measurements along the visual axis. The measured parameters include anterior chamber depth (ACD), lens thickness (LENS), and axial length (AXIAL). **b** Quantification of total retinal thickness measured from WT and *Prss56* KI mice. Retinal thickness was measured on representative H&E-stained retinal

sections using the optic nerve head as the anatomical reference; *n* = 3 mice for each genotype. **c** Identification of genotype of *Prss56* knockout mice. **d**, Decreased PRSS56 protein level in eye tissue of *Prss56* knockout mice compared with WT. **e** Genotyping strategy for F1 offspring of PRSS56-Tet-on transgenic mice. **f** Identification of genotype of PRSS56-Tet-on mice. **g** Robust PRSS56 upregulation occurred exclusively in DOX-induced Tet-on mice, with no significant increase observed in either DOX-treated WT or uninduced homozygous Tet-on controls. **h** Schematic representation of AAV2 viruses were individually injected into the vitreous cavities of 1 day-old mice. **i, j** AAV2 efficiency (AAV2-eGFP and AAV2-$PRSS56^{WT}$) was evaluated by immunofluorescence (**i**) and western blotting (**j**) for the stretched retina of mice at postnatal days 14. Scale bar, 50 μm and 200 μm.
