# Supplementary material for: Increased PRSS56 expression is a causal factor and therapeutic target for human axial high myopia
Source: Cell Res. 2026 Apr 1;36(8):567–81. doi: 10.1038/s41422-026-01241-9 (PMC13424129; doi:10.1038/s41422-026-01241-9)
Supplement: Supplementary file 5 — Supplementary Information, Fig. S5 [file 41422_2026_1241_MOESM5_ESM.pdf]

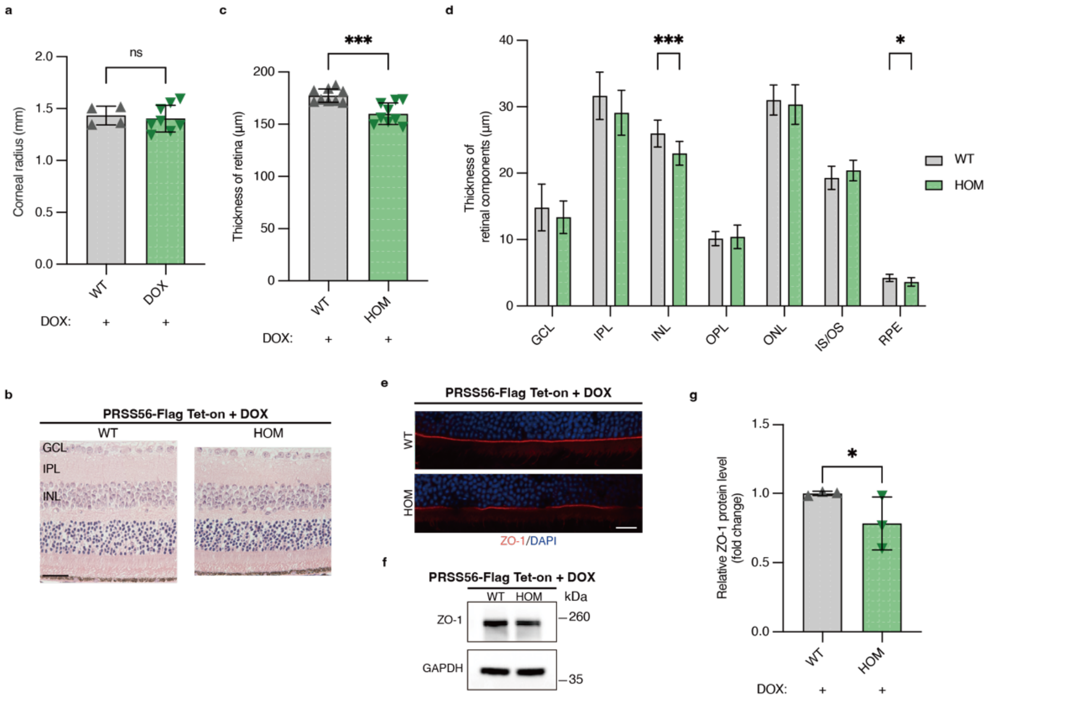

Supplementary information, Fig. S5 Retinal effects of PRSS56 overexpression

a Corneal radius of curvature measured by SD-OCT shows no significant difference between doxycycline treated Tet-on mice and controls; n = 4-8 mice for each genotype. **b** HE staining of the retina showed decreased retinal thickness of the PRSS56-Tet-on HOM mice compared with WT. Scale bar, 20 μm. **c** Retinal thickness quantification revealed significant thinning in DOX-induced Tet-on mice; *n* = 9 mice for each genotype. **d** Layer-specific thickness distribution in retinas. The INL and RPE exhibited significant thinning in DOX-induced Tet-on mice; *n* = 9 mice for each genotype. **e** Immunofluorescence revealed significantly reduced ZO-1 expression at the external limiting membrane (ELM) in DOX-induced Tet-on retinas. Scale bar, 20 μm. **f** Western blot analysis demonstrated significantly decreased protein levels of ZO-1 in ocular tissues of DOX-induced Tet-on mice. **g** Quantification of ZO-1 protein levels normalized to GAPDH; *n* = 3 mice for each genotype.
