# Supplementary material for: Increased PRSS56 expression is a causal factor and therapeutic target for human axial high myopia
Source: Cell Res. 2026 Apr 1;36(8):567–81. doi: 10.1038/s41422-026-01241-9 (PMC13424129; doi:10.1038/s41422-026-01241-9)
Supplement: Supplementary file 6 — Supplementary Information, Fig. S6 [file 41422_2026_1241_MOESM6_ESM.pdf]

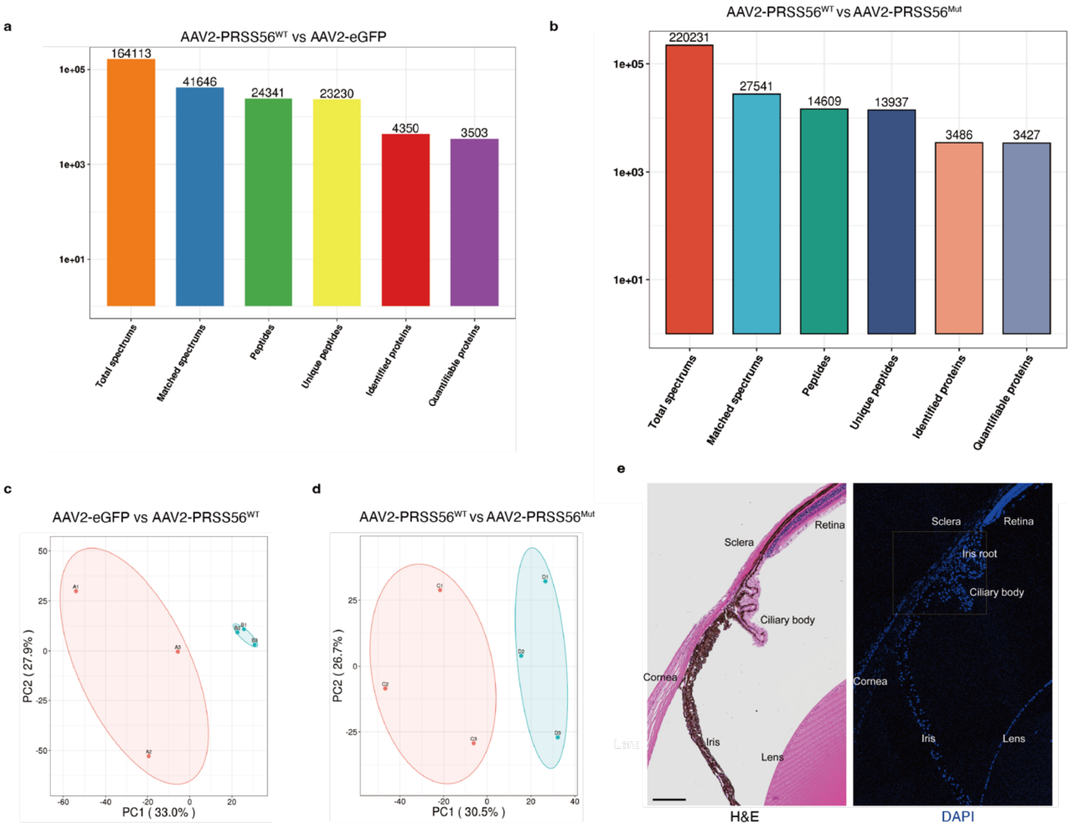

Supplementary information, Fig. S6 Proteomic profiling of AAV2-injected mouse eyes

a-d Quantitative proteomic analysis of mouse eyes injected with AAV2-eGFP versus AAV2-PRSS56^{WT} (n = 3) as group 1, and AAV2-PRSS56^{WT} versus AAV2-PRSS56^{Mut} (n = 3) as group 2. Overview of quantitative proteomic analysis of group 1 (**a**) and group 2 (**b**). Proteomic principal component analysis (PCA) of group 1 (**c**) and group 2 (**d**), respectively. **e** Representative consecutive sections showing histological staining and localization of ocular substructures in mouse eyes. Major ocular substructures are indicated. The yellow box denotes the ciliary region and serves as a reference for orientation in subsequent immunofluorescence analyses. Scale bar, 100 µm.
