# Supplementary material for: Increased PRSS56 expression is a causal factor and therapeutic target for human axial high myopia
Source: Cell Res. 2026 Apr 1;36(8):567–81. doi: 10.1038/s41422-026-01241-9 (PMC13424129; doi:10.1038/s41422-026-01241-9)
Supplement: Supplementary file 7 — Supplementary Information, Fig. S7 [file 41422_2026_1241_MOESM7_ESM.pdf]

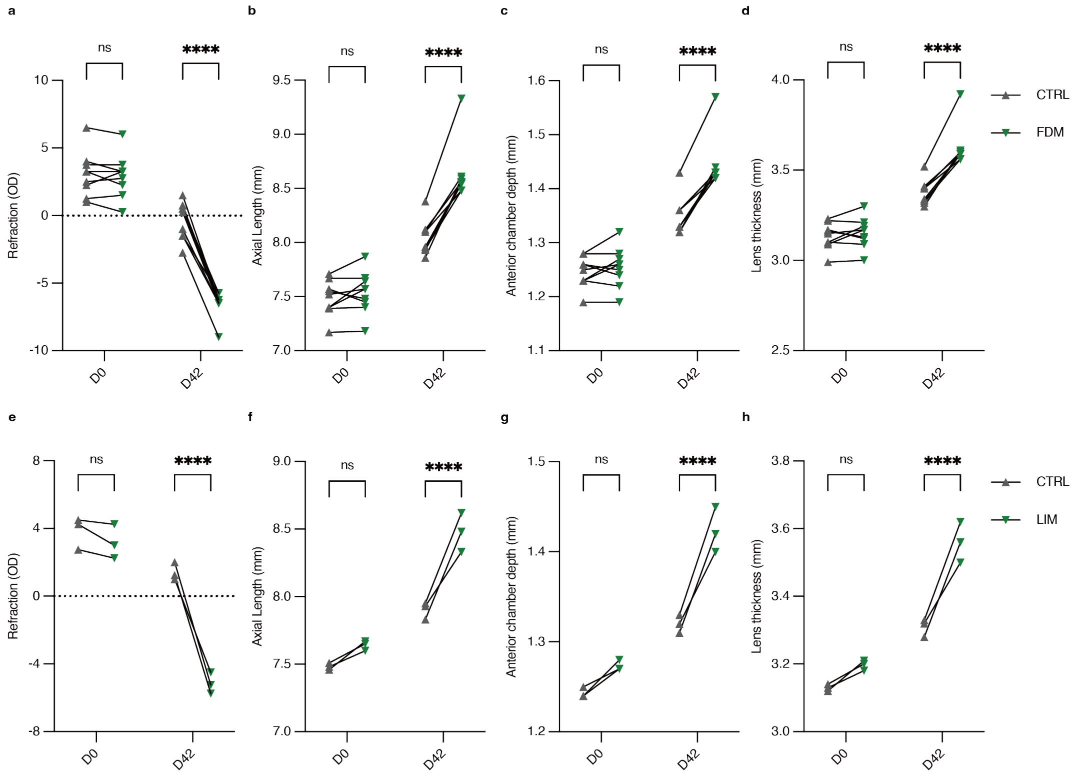

Supplementary information, Fig. S7 Biometric and refractive outcomes in FDM and LIM models

Biometric and refractive outcomes after 42 days of treatment. **a-d** show results from the FDM model and **e-h** show results from the LIM model. For each model the four panels present refractive error (**a** and **e**), axial length (**b** and **f**), anterior chamber depth (**c** and **g**) and lens thickness (**d** and **h**) measured in treated eyes and contralateral controls; *n* = 3-9 guinea pigs for each condition.
