# Supplementary material for: Increased PRSS56 expression is a causal factor and therapeutic target for human axial high myopia
Source: Cell Res. 2026 Apr 1;36(8):567–81. doi: 10.1038/s41422-026-01241-9 (PMC13424129; doi:10.1038/s41422-026-01241-9)
Supplement: Supplementary file 8 — Supplementary Information, Fig. S8 [file 41422_2026_1241_MOESM8_ESM.pdf]

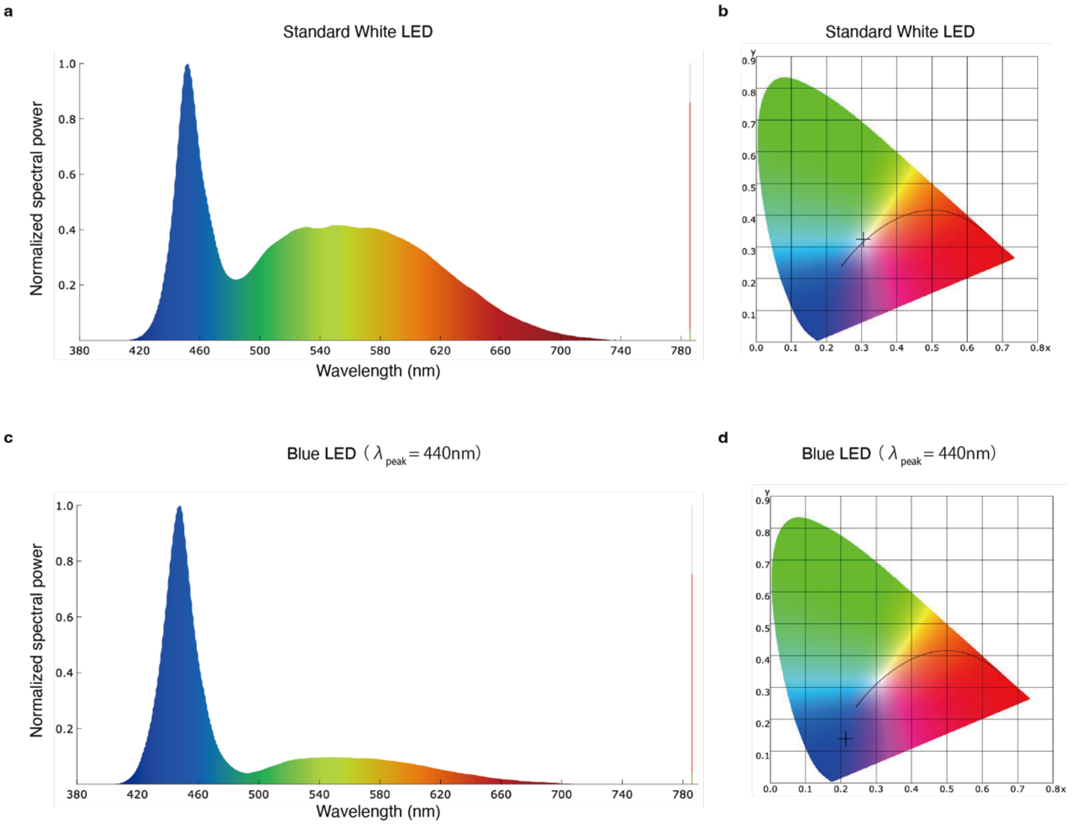

Supplementary information, Fig. S8 Characterization of white and blue LED light

a Relative spectral power distribution of a standard white LED. **b** The x-y chromaticities plotted on CIE 1931 color space of the white LED. **c** Relative spectral power distribution of the blue LED source. **d** The x-y chromaticities plotted on CIE 1931 color space of the blue light.
