# Supplementary material for: Increased PRSS56 expression is a causal factor and therapeutic target for human axial high myopia
Source: Cell Res. 2026 Apr 1;36(8):567–81. doi: 10.1038/s41422-026-01241-9 (PMC13424129; doi:10.1038/s41422-026-01241-9)
Supplement: Supplementary file 9 — Supplementary Information, Fig. S9 [file 41422_2026_1241_MOESM9_ESM.pdf]

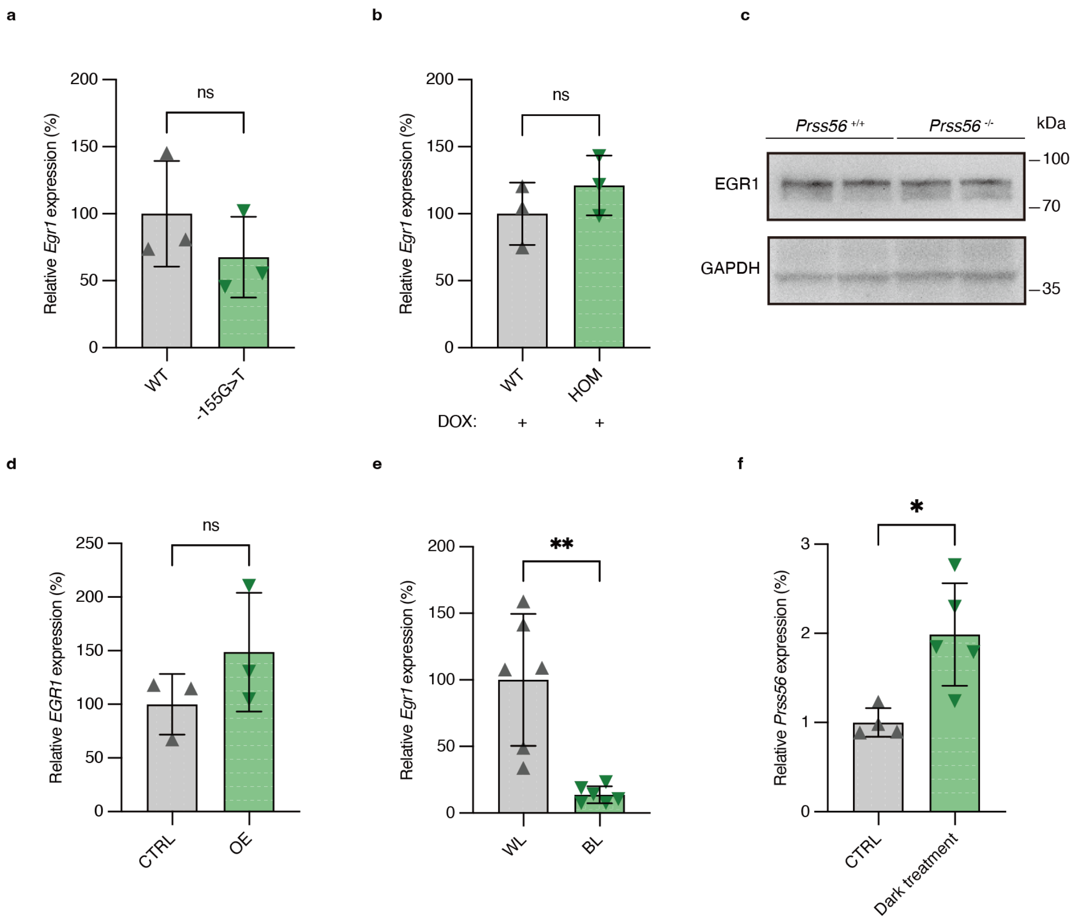

Supplementary information, Fig. S9 *EGR1/Egr1* expression under PRSS56 modulation and light conditions

a *Egr1* transcript levels in retinas from *Prss56* KI mice and matched WT controls did not differ significantly; $n = 3$ mice for each genotype. **b** *Egr1* transcript levels in retinas from doxycycline treated Tet-on mice and matched controls did not differ significantly; $n = 3$ mice for each genotype. **c** EGR1 protein levels in whole-eye lysates from *Prss56* KO mice and WT controls showed no significant difference; $n = 3$ mice for each genotype. **d** *EGR1* expression in MIO-M1 cells after *PRSS56* overexpression compared with empty vector controls did not differ significantly; $n = 3$ biological replicates for condition. **e** *Egr1* expression is significantly reduced in BALB/c mice after short-wave light (blue, 417nm) exposure; $n = 6$ mice for each condition . **f** qPCR analysis in *Prss56*

KI mice under dark rearing show significant upregulation of *Prss56* expression in the mouse retina. The Dark Treatment group was reared in complete darkness from birth, while the Control group experienced a normal light-dark cycle. Expression levels were assessed at P11; n = 4-5 mice for each genotype.
