# Supplementary material for: Increased PRSS56 expression is a causal factor and therapeutic target for human axial high myopia
Source: Cell Res. 2026 Apr 1;36(8):567–81. doi: 10.1038/s41422-026-01241-9 (PMC13424129; doi:10.1038/s41422-026-01241-9)
Supplement: Supplementary file 10 — Supplementary Information, Table S1 [file 41422_2026_1241_MOESM10_ESM.pdf]

Supplementary information, Table S1

Clinical and genetic characteristics of F1 and F2. U, unaffected; A, affected.

Individual	Sex	Age	Affected status	Refractive error (Ds)		Intraocular pressure (mmHg)		Axial length (mm)		Mutation
				OD	OS	OD	OS	OD	OS	
F1-I-1	M	78	U	+01.63	+00.38	14.5	17.9	22.57	22.42	c.-187 G
F1-I-2	F	80	A	N.a	N.a	N.a	N.a	N.a	N.a	c.-187 G > T
F1-II-1	M	50	U	+00.13	-00.13	10.7	11.0	21.57	21.45	c.-187 G
F1-II-2	F	48	A	-18.88	-20.88	22.2	20.4	33.22	32.58	c.-187 G > T
F1-II-3	F	52	U	+00.13	+00.25	17.6	19.3	22.54	22.63	c.-187 G
F1-II-4	M	57	A	-14.88	-16.25	15.1	14.8	33.77	32.20	c.-187 G > T
F1-II-5	F	54	U	-01.13	-00.75	18.2	13.9	22.48	22.38	c.-187 G
F1-II-6	M	43	U	+00.25	-00.25	15.5	14.4	24.11	23.80	c.-187 G
F1-II-7	F	37	U	Plano	Plano	15.4	15.5	22.30	22.29	c.-187 G
F1-II-8	F	40	A	-20.38	-20.88	13.2	17.9	33.55	34.18	c.-187 G > T
F1-II-9	M	40	U	Plano	Plano	12.1	16.5	22.97	22.97	c.-187 G
F1-III-1	M	20	A	-12.88	-13.50	17.9	19.0	27.74	29.79	c.-187 G > T
F1-III-2	F	23	U	Plano	Plano	14.0	14.5	23.01	22.69	c.-187 G
F1-III-3	M	39	U	Plano	Plano	15.6	17.4	22.67	22.65	c.-187 G
F1-III-4	F	32	A	-18.75	-18.75	22.3	18.4	33.31	31.36	c.-187 G > T
F1-III-5	F	30	A	-18.63	-09.88	22.4	19.2	26.36	31.34	c.-187 G > T
F1-III-6	M	29	U	Plano	Plano	10.7	11.8	22.51	22.80	c.-187 G
F1-III-7	M	15	U	Plano	Plano	15.4	15.6	24.37	24.49	c.-187 G
F1-III-8	F	6	A	-16.38	-16.75	15.8	15.5	29.35	29.05	c.-187 G > T
F1-IV-1	M	2	A	-05.38	-05.13	N.a	N.a	N.a	N.a	c.-187 G > T
F1-IV-3	M	5	A	-07.00	-08.13	14.5	14.8	25.53	25.68	c.-187 G > T
F2-II-1	M	61	U	+01.50	+01.50	16.7	15.8	22.15	22.21	c.-187 G
F2-II-2	F	59	A	-09.88	-04.13	12.7	11.5	31.23	30.27	c.-187 G > C
F2-II-3	M	46	A	-15.00	-15.00	17.8	16.4	29.88	29.67	c.-187 G > C
F2-II-4	F	45	U	Plano	Plano	12.5	13.2	23.12	23.04	c.-187 G
F2-III-1	F	30	U	-00.50	-00.50	16.6	15.6	22.87	22.96	c.-187 G
F2-III-3	M	32	A	N.a	-17.75	5.0	14.2	N.a	33.38	c.-187 G > C
F2-III-4	F	31	U	-01.00	-01.00	14.6	15.8	23.45	25.68	c.-187 G
F2-III-5	F	18	U	-01.00	-01.00	14.6	15.8	23.45	25.68	c.-187 G
F2-IV-1	F	7	U	Plano	Plano	12.7	13.4	23.13	22.98	c.-187 G
F2-IV-2	M	4	A	-17.50	-17.00	16.8	18.0	29.56	29.51	c.-187 G > C
