# Supplementary material for: Increased PRSS56 expression is a causal factor and therapeutic target for human axial high myopia
Source: Cell Res. 2026 Apr 1;36(8):567–81. doi: 10.1038/s41422-026-01241-9 (PMC13424129; doi:10.1038/s41422-026-01241-9)
Supplement: Supplementary file 11 — Supplementary Information, Table S2 [file 41422_2026_1241_MOESM11_ESM.pdf]

Supplementary information, Table S2

CNVs identified by aCGH.

Location	Size (bp)	Loci	CNV type	Gene
Chr1:72241965-72,251552	9588	1p31.1	loss	*NEGR1*
Chr2:28416180-28423492	7313	2p35.3	loss	*BRE*
Chr2:233972680-233978178	5499	2q37.1	gain	*INPP5D*
Chr2:233984174-233987023	2850	2q37.1	loss	*INPP5D*
Chr2:232572000-232574738	2739	2q37.1	loss	*PTMA*
Chr4:93708270-93777324	69055	4q22.1	loss	*GRID2*
Chr5:174862802-174874489	11688	5q35.2	loss	*DRD1*
Chr7:51345798-51359026	13229	7p12.1	loss	*COBL*
Chr10:127945210-127958705	13496	10q26.2	gain	*ADAM12*
