# Supplementary material for: Increased PRSS56 expression is a causal factor and therapeutic target for human axial high myopia
Source: Cell Res. 2026 Apr 1;36(8):567–81. doi: 10.1038/s41422-026-01241-9 (PMC13424129; doi:10.1038/s41422-026-01241-9)
Supplement: Supplementary file 12 — Supplementary Information, Table S3 [file 41422_2026_1241_MOESM12_ESM.pdf]

Supplementary information, Table S3

Statistics for whole exome sequencing.

Coverage Analysis	F1-II-2	F1-III-8	F1-IV-1	F1-IV-3	F1-III-3	F1-III-6
Mapped Reads	84257968	95699403	121031718	139057808	110518654	125014833
Total effective reads	83068632	94051030	90585579	104441723	82493528	93560535
Mean Depth in Target	94.74	110.48	134.39	152.64	126.44	140.55
Coverage of target region(%)	97.31%	97.55%	96.02%	96.27%	96.04%	96.21%
Fraction of target covered with at least 1X(%)	99.76%	99.83%	99.90%	99.87%	99.89%	99.85%
Fraction of target covered with at least 10X(%)	98.95%	99.12%	98.47%	99.15%	98.95%	99.07%
Fraction of target covered with at least 30X(%)	92.31%	94.36%	92.06%	94.97%	92.24%	94.28%
