# Supplementary material for: Increased PRSS56 expression is a causal factor and therapeutic target for human axial high myopia
Source: Cell Res. 2026 Apr 1;36(8):567–81. doi: 10.1038/s41422-026-01241-9 (PMC13424129; doi:10.1038/s41422-026-01241-9)
Supplement: Supplementary file 13 — Supplementary Information, Table S4 [file 41422_2026_1241_MOESM13_ESM.pdf]

Supplementary information, Table S4

Statistics for whole genome sequencing.

Coverage Analysis	F1-II-2	F1-III-1
Total Reads(M)	1410.66	1370.13
Mapping Rate	99.81%	99.36%
Total effective yield(Gb)	201.58	196.92
Mean coverage sequencing depth	66.5	63.8
Coverage of target region(%)	92.93%	91.28%
Fraction of target covered with at least 30X(%)	90.96%	89.78%
