# Supplementary material for: Increased PRSS56 expression is a causal factor and therapeutic target for human axial high myopia
Source: Cell Res. 2026 Apr 1;36(8):567–81. doi: 10.1038/s41422-026-01241-9 (PMC13424129; doi:10.1038/s41422-026-01241-9)
Supplement: Supplementary file 14 — Supplementary Information, Table S5 [file 41422_2026_1241_MOESM14_ESM.pdf]

Supplementary information, Table S5

Ocular biometric and genotype of the sporadic patient cohort.

ID	RE (Ds)		AL (mm)		GT	ID	RE (Ds)		AL (mm)		GT
	OD	OS	OD	OS			OD	OS	OD	OS	
HM-6	-06.00	+00.50	25.35	22.81	WT	HM-820	-24.00	-27.00	27.63	27.77	WT
HM-9	-12.50	-11.75	27.9	27.81	WT	HM-821	-09.50	-09.50	25.84	25.65	WT
HM-13	-07.50	-07.50	25.76	26.23	WT	HM-823	-12.00	-12.75	27.45	27.54	WT
HM-29	+00.50	-16.00	22.37	28.25	WT	HM-823	-13.25	-12.75	27.94	28.00	WT
HM-30	-05.50	-09.50	23.59	24.88	WT	HM-825	-10.00	-09.00	26.42	25.81	WT
HM-33	-15.00	-16.00	29.27	29.42	WT	HM-827	+01.00	-09.00	22.52	26.16	WT
HM-37	-11.00	-05.25	27.28	25.06	WT	HM-828	-07.75	-07.75	25.57	25.72	WT
HM-38	-07.50	+00.50	24.59	22.18	WT	HM-831	-05.50	-07.75	25.03	26.16	WT
HM-156	-11.50	-13.25	28.05	28.75	WT	HM-833	-09.00	-08.50	27.35	27.36	WT
HM-157	-10.50	-08.00	27.60	27.80	WT	HM-837	-10.00	-10.75	27.05	26.94	WT
HM-158	-06.50	-05.00	23.87	23.66	WT	HM-839	-09.38	-07.75	25.34	24.75	WT
HM-159	-06.25	-07.25	25.50	25.57	WT	HM-840	-13.50	-11.75	25.80	25.99	WT
HM-170	-08.00	-08.50	25.48	25.34	WT	HM-841	-08.88	-09.50	26.01	26.12	WT
HM-171	-04.50	-09.00	25.65	27.23	WT	HM-842	-12.75	-14.25	28.07	28.36	c.-382C>T
HM-179	-09.50	-10.00	27.27	27.52	WT	HM-846	-00.25	-08.38	23.77	26.86	WT
HM-180	-10.00	-03.50	27.53	25.02	WT	HM-847	+00.75	-15.38	21.59	26.42	WT
HM-181	-01.50	-08.00	23.09	26.45	WT	HM-848	-09.63	-16.50	25.90	25.72	WT
HM-183	-11.50	-12.00	26.17	25.97	c.-382C>T	HM-849	-07.50	-08.88	25.48	25.78	WT
HM-195	-15.00	+01.00	29.25	23.20	WT	HM-867	-09.25	-08.75	26.13	26.01	WT
HM-196	-13.75	-15.00	27.16	27.57	WT	HM-868	-13.00	-13.25	26.17	26.13	WT
HM-198	-06.00	-08.00	25.29	25.86	WT	HM-869	N.a	-08.25	22.22	24.88	WT
HM-199	-06.75	-05.75	26.09	25.87	WT	HM-870	-09.00	-08.63	27.25	26.84	WT
HM-301	-06.00	-07.50	26.51	27.07	WT	HM-871	-14.25	-12.25	28.37	27.34	WT
HM-303	-07.50	-05.50	25.86	25.00	WT	HM-872	-14.25	-15.25	27.53	27.93	WT
HM-304	-07.25	-08.50	23.92	24.69	WT	HM-873	-09.00	-02.63	25.23	22.64	WT
HM-305	-06.00	-06.50	26.07	26.15	WT	HM-875	-08.88	-08.38	26.64	26.29	WT
HM-308	-15.00	-12.00	29.23	28.15	WT	HM-876	-06.63	-07.38	24.82	24.89	WT
HM-310	-07.25	-07.25	26.48	26.35	WT	HM-877	-09.75	-10.50	25.43	25.57	WT
HM-318	-09.00	-10.50	24.90	25.20	WT	HM-878	-08.50	-07.63	26.03	25.97	WT
HM-320	-14.50	-00.75	27.14	22.18	WT	HM-879	-06.38	-06.13	24.22	24.28	WT
HM-325	-12.00	-11.00	28.79	28.43	WT	HM-880	-12.50	-11.75	25.92	25.83	WT
HM-326	-10.00	-13.00	27.78	27.94	WT	HM-881	-09.00	-09.63	24.3	24.79	WT
HM-327	-12.00	-12.00	26.79	26.77	WT	HM-882	-08.63	-08.88	25.96	26.08	WT
HM-328	+00.50	-13.00	23.32	28.64	WT	HM-883	-07.75	-07.38	25.45	25.3	WT
HM-331	-19.00	+01.00	27.61	21.54	WT	HM-884	-07.25	-09.38	25.02	25.72	WT
HM-387	-08.00	-02.00	26.05	23.92	WT	HM-888	-06.25	-05.13	25.73	25.30	WT
HM-388	-05.00	-05.50	N.a	N.a	WT	HM-889	-11.50	-11.00	26.52	26.65	WT
HM-391	-11.50	-10.50	25.77	25.44	WT	HM-890	-10.75	-11.13	25.69	26.00	WT

HM-395	-07.75	-10.50	25.70	26.65	WT	HM-892	-11.50	+0.75	26.36	22.03	WT
HM-396	-12.00	-12.00	28.70	29.15	WT	HM-893	-08.50	-08.38	25.21	24.96	WT
HM-397	-13.00	-10.00	27.58	26.53	WT	HM-895	-06.88	-08.25	24.85	25.23	WT
HM-398	-18.00	-18.00	28.90	28.72	WT	HM-896	-07.88	-10.50	23.64	25.06	WT
HM-399	-13.75	-10.50	26.82	25.77	WT	HM-901	-15.63	-13.63	28.10	27.17	WT
HM-406	-10.00	-10.00	26.62	26.42	WT	HM-902	-18.13	-18.88	28.73	28.61	WT
HM-407	-05.50	-06.00	25.52	26.12	WT	HM-903	-11.00	-03.25	24.68	22.56	WT
HM-408	-08.00	-06.50	26.21	25.44	WT	HM-904	-11.75	-11.25	26.28	26.36	WT
HM-411	-09.00	-08.00	26.43	25.95	WT	HM-905	-10.25	-09.00	26.63	26.29	WT
HM-412	-13.50	-13.00	27.82	27.50	WT	HM-906	-13.00	-13.38	27.41	27.38	WT
HM-415	-13.00	-08.25	27.49	26.60	WT	HM-907	-19.50	-01.25	27.39	21.91	WT
HM-416	-10.50	-10.00	26.60	26.78	WT	HM-908	-08.00	-03.63	24.33	23.05	WT
HM-418	-08.50	-08.25	26.93	26.51	WT	HM-910	-16.50	-16.50	30.40	30.47	c.-421G>A
HM-419	-07.50	-06.00	24.88	24.55	WT	HM-915	-06.88	N.a	25.56	22.84	WT
HM-453	-13.75	-13.75	27.12	27.61	WT	HM-916	-09.75	-10.25	26.07	26.40	WT
HM-454	N.a	-12.00	N.a	24.22	WT	HM-917	N.a	-09.00	21.89	25.19	WT
HM-455	+01.25	-10.50	21.74	25.99	WT	HM-918	-00.75	-09.38	22.56	26.18	WT
HM-457	-08.00	-09.00	24.77	25.36	WT	HM-919	-15.13	-09.50	29.56	27.54	WT
HM-460	-06.50	-08.00	25.05	25.03	WT	HM-920	-08.75	-08.63	24.49	24.66	WT
HM-461	-08.50	+00.50	23.18	20.77	WT	HM-921	-05.63	-18.75	25.07	29.15	WT
HM-462	-06.75	-07.00	25.09	25.24	WT	HM-923	-09.00	-12.88	27.35	28.08	WT
HM-463	-11.50	-11.75	26.39	26.15	WT	HM-925	-10.88	-12.75	27.01	27.35	WT
HM-465	-12.00	-11.00	26.42	26.12	WT	HM-928	-01.25	-20.25	23.33	29.35	WT
HM-467	-10.00	-10.00	26.70	26.54	WT	HM-929	-00.38	-11.38	22.09	24.88	WT
HM-468	-04.75	-05.75	26.01	26.55	WT	HM-931	-17.13	-18.50	29.75	30.11	WT
HM-469	-07.00	-03.00	26.42	24.70	WT	HM-932	-12.00	-08.50	27.77	26.88	WT
HM-470	-12.00	-11.00	26.48	26.44	WT	HM-934	-13.38	-09.13	28.01	26.37	WT
HM-471	-06.25	-06.50	24.43	24.74	WT	HM-1115	-11.88	-14.00	24.96	25.94	WT
HM-475	-04.00	-10.00	23.38	25.97	WT	HM-1116	-12.75	-11.75	29.47	29.17	WT
HM-476	-05.25	-09.00	23.55	24.87	WT	HM-1117	-09.38	-09.25	25.96	26.07	WT
HM-477	-06.50	-07.25	25.77	25.70	WT	HM-1120	-12.25	-13.50	26.29	26.53	WT
HM-481	-09.00	-10.25	24.71	25.23	WT	HM-1122	-13.25	-01.63	25.93	22.32	WT
HM-482	-09.00	-08.00	25.56	25.04	WT	HM-1124	-13.75	N.a	27.89	28.27	WT
HM-487	-08.25	-01.50	25.98	23.26	WT	HM-1125	N.a	-08.50	28.55	27.90	WT
HM-488	-06.50	-07.50	24.95	25.29	WT	HM-1127	-07.50	-08.25	26.41	25.20	WT
HM-489	-13.00	-13.00	28.17	28.04	WT	HM-1129	-09.50	-01.75	25.19	25.24	WT
HM-491	-10.50	N.a	25.00	21.78	WT	HM-1132	N.a	-11.00	25.11	22.83	WT
HM-492	-12.00	-11.00	25.48	25.37	WT	HM-1133	-11.00	-09.25	21.89	25.68	WT
HM-493	-10.50	-08.50	28.56	27.77	WT	HM-1135	N.a	-09.00	27.75	27.42	WT
HM-494	+00.50	-09.75	23.76	26.85	WT	HM-1136	-06.88	+06.00	23.27	26.47	WT
HM-495	-14.00	+00.50	27.62	22.41	WT	HM-1137	-13.00	-11.00	25.29	25.21	c.-378G>A
HM-496	-21.00	-09.00	27.45	24.63	WT	HM-1138	-09.50	-08.13	26.80	26.35	WT
HM-498	-07.00	-06.00	25.65	25.41	WT	HM-1139	-07.75	-10.88	25.39	24.76	WT

HM-499	-13.25	-13.50	29.30	28.97	WT	HM-1141	-07.25	-06.50	25.97	26.83	WT
HM-504	-00.25	-12.50	22.15	26.25	WT	HM-1143	-06.00	-04.88	25.53	25.04	WT
HM-508	-07.75	-07.50	25.97	26.14	WT	HM-1144	-18.25	+01.00	23.93	23.55	WT
HM-510	-05.00	-06.00	25.6	25.85	WT	HM-1146	-12.63	-10.38	26.34	22.19	WT
HM-511	-19.50	-12.00	27.42	25.01	WT	HM-1147	-21.50	-20.25	26.71	25.90	WT
HM-513	-09.50	-08.50	24.23	23.74	WT	HM-1150	-13.75	-13.75	30.04	29.44	WT
HM-514	-12.50	-13.50	28.18	29.05	WT	HM-1151	-15.88	-00.50	29.25	28.97	WT
HM-515	-09.50	-08.75	26.46	26.42	WT	HM-1152	-07.63	-09.25	27.46	22.98	WT
HM-518	-05.75	-05.75	24.60	24.38	WT	HM-1153	-23.50	-17.00	25.97	26.42	WT
HM-519	-07.00	-06.00	26.85	26.15	WT	HM-1156	-11.63	-11.13	29.35	27.76	WT
HM-524	+01.50	-12.00	22.71	27.60	WT	HM-1157	-06.00	-09.25	26.51	26.35	WT
HM-525	-13.00	-14.50	27.2	27.45	c.-187G>C	HM-1159	-11.25	+00.25	25.33	26.31	WT
HM-527	-15.50	+00.25	29.48	23.99	WT	HM-1161	-08.50	-10.50	26.03	22.59	WT
HM-528	-14.00	-15.75	26.82	27.47	WT	HM-1162	+00.50	-15.75	26.83	27.12	WT
HM-530	-21.00	-21.00	31.38	31.91	WT	HM-1163	-00.75	-07.88	22.12	27.42	WT
HM-532	-13.00	-13.00	29.26	29.4	WT	HM-1164	-07.00	-09.50	22.91	25.75	WT
HM-534	-10.00	-11.00	25.22	25.61	WT	HM-1165	-09.63	-08.75	24.90	26.23	WT
HM-536	-06.00	-05.25	24.66	24.2	WT	HM-1168	-06.63	-07.25	28.47	28.04	WT
HM-554	-08.00	-08.50	25.95	25.78	WT	HM-1169	-14.00	-13.00	24.72	24.79	WT
HM-579	-06.25	-05.50	26.13	25.67	WT	HM-1173	-05.25	-09.75	28.85	28.64	WT
HM-616	-13.75	-13.25	25.65	25.58	WT	HM-1175	-05.38	-09.00	24.04	25.18	WT
HM-634	-17.50	-17.50	34.66	35.04	WT	HM-1177	-06.88	-08.00	24.77	25.83	WT
HM-657	-09.50	-12.50	24.93	25.25	WT	HM-1178	-09.25	-09.13	24.95	25.08	WT
HM-786	-07.50	-07.00	26.26	25.91	WT	HM-1179	-11.00	-10.63	25.12	24.56	WT
HM-787	-07.25	-08.00	28.12	28.45	WT	HM-1180	-06.25	-07.13	28.30	28.15	WT
HM-788	-21.00	-21.00	31.05	31.18	WT	HM-1181	-11.75	-14.88	25.12	25.23	c.-297C>T
HM-790	-09.50	-06.50	27.86	25.84	WT	HM-1183	-10.50	-06.88	25.41	26.41	WT
HM-804	-20.00	-21.50	33.86	33.07	WT	HM-1184	-07.13	-10.38	25.38	24.28	WT
HM-806	-11.00	-11.50	26.43	26.20	WT	HM-1187	-09.75	-04.00	24.71	25.92	WT
HM-807	-12.50	+01.25	26.06	22.84	WT	HM-1188	-11.75	-09.13	26.49	24.64	WT
HM-808	-02.25	-06.00	24.19	25.31	WT	HM-1196	-13.00	-06.38	26.03	25.13	WT
HM-809	-10.25	-09.00	26.74	26.56	WT	HM-1197	-13.38	-12.25	27.85	24.85	WT
HM-812	-17.00	-16.00	29.56	29.51	WT	HM-1198	-10.00	+01.25	29.46	29.15	WT
HM-813	-05.75	-06.75	25.86	25.82	WT	HM-1199	-08.25	-09.13	24.16	21.05	WT
HM-815	-14.50	+01.00	26.66	22.24	WT	HM-1200	-08.13	-08.13	25.46	25.55	WT
HM-816	-06.00	-06.25	24.71	24.55	WT	HM-1201	-18.25	-11.88	24.25	24.59	WT
HM-819	-07.00	-07.00	25.14	25.18	WT	HM-1202	-07.75	-07.38	29.10	27.01	WT
