# Supplementary material for: Increased PRSS56 expression is a causal factor and therapeutic target for human axial high myopia
Source: Cell Res. 2026 Apr 1;36(8):567–81. doi: 10.1038/s41422-026-01241-9 (PMC13424129; doi:10.1038/s41422-026-01241-9)
Supplement: Supplementary file 15 — Supplementary Information, Table S6 [file 41422_2026_1241_MOESM15_ESM.pdf]

Supplementary information, Table S6

Primers and sgRNA used in this study.

Primers	Sequences（5'-3'）
hPRSS56-seqF	CCAGAAGAATCCCACTGCCAG
hPRSS56-seqR	GGGTGCCTATCCTTTGGTGC
PGL3-PRSS56-proF	CTAGCTAGCGCGCTAATTAGGCTGCTTTG
PGL3-PRSS56-proR	CCCAAGCTTGGGCACTGAATTCTTGTCCC
-187G>T-F	AGGAGTTAAGGGCCAGTTGAGGGCTGACCAGGG
-187G>T-R	CCCTGGTCAGCCCTCAACTGGCCCTTAACTCCT
-187G>C-F	AGGAGTTAAGGGCCAGCTGAGGGCTGACCAGGG
-187G>C-R	CCCTGGTCAGCCCTCAGCTGGCCCTTAACTCCT
-297C>T-F	AAGCCCACTACCCTACTCCTCCCCCAGTCTTGGC
-297C>T-R	AGTAGGGTAGTGGGCTTGACCTTCACCTCTGA
-378G>A-F	GAGCCGCAAAAACAAGGGCTCCTTTGACAGAA
-378G>A-R	CCTTGTTTTTGCGGCTCCATCAAAGGGGCCAA
-382C>T-F	GATGGAGCTGCAGAAACAAGGGCTCCTTTGAC
-382C>T-R	GTTTCTGCAGCTCCATCAAAGGGGCCAAGACA
-421G>A-F	CAACCATCAGCCCTTCTTCCCTGTCTTGGCCC
-421G>A-R	AGAAGGGCTGATGGTTGGGCTGCAGGCCAGGC
hEGR1-F	AGGAGATCTGCCGCCGCGATCGCATGGCCGCGGCCAAGGCC
hEGR1-R	CTCGAGCGGCCGCGTACGCGTGCAAATTTCAATTGTCCTGGGA
Gprobe-F	TAAGGGCCAGGTGAGGGCTGACCAGG
Gprobe-R	CCTGGTCAGCCCTCACCTGGCCCTTA
Tprobe-F	TAAGGGCCAGTTGAGGGCTGACCAGG
Tprobe-R	CCTGGTCAGCCCTCAACTGGCCCTTA
Cprobe-F	TAAGGGCCAGCTGAGGGCTGACCAGG
Cprobe-R	CCTGGTCAGCCCTCAGCTGGCCCTTA
SgRNA (KI model)	CTAATGGTCCGCACCTCACC
ssODN	GGCTGCCCTCCCACGCACACACACCCCTCTGTCCTCTATATAGCTCCAGGGCATCGTTTCCCTGAGGGC<br>TCCCAGGAGGTGAGGGCCAGtTGAGGTGCGGACCATTAGAAGTGAGTAGTTTAGTGTAAG
KI-genoty F	GTGGCAGAAACAGATGGGGA
KI-genoty R	CAGTTTGGGAGTCTGGGGAC
KO-genoty F	CCAGACAAGGTTCAGGAAGACT
KO-genoty R	ATGAACTGTGACCACCTGAGC
SgRNA (Tet-on model)	CTGAGCCAACAGTGGTAGTA
Tet-on-genoty F1	AGTCTTTCCCTTGCCTCTGCT
Tet-on-genoty R1	GGGTCTTCCACCTTTCTTCAG
Tet-on-genoty F2	GTGTAAGTCCTTTCAAAGGGCTCC
Tet-on-genoty R2	ATATCCCCTTGTTCCCTTTCTGC
hPRSS56-qPCR-F	ACTGTACACACGCCTGCC
hPRSS56-qPCR-F	CAGATCCTCGGCACTCGT
mPrss56-qPCR-F	TGTGGACTGTGATGCTCGCC
mPrss56-qPCR-R	GAACCCTGGGGAAGGCAAAT
gpPRSS56-qPCR-F	AGCACCCTGCAAGTTCTGTC

gpPRSS56-qPCR-F	AGGTCCTCGACACTCATGGA
mMyh4-qPCR-F	TTGAAAAGACGAAGCAGCGAC
mMyh4-qPCR-R	AGAGAGCGGGACTCCTTCTG
mCol1a1-qPCR-F	GCTCCTCTTAGGGGCCACT
mCol1a1-qPCR-R	ATTGGGGACCCTTAGGCCAT
hGAPDH-qPCR-F	GAAGGTGAAGGTCGGAGTC
hGAPDH-qPCR-R	GAAGATGGTGATGGGATTTC
mGapdh-qPCR-F	TAACATCAAATGGGGTGAGG
mGapdh-qPCR-R	GGTTCACACCCATCACAAAC
gpGapdh-qPCR-F	ATCAAGTGGGGTGATGCTGG
gpGapdh-qPCR-R	TTTGGCACCACCCTTCAAGT
