# Supplementary material for: Increased PRSS56 expression is a causal factor and therapeutic target for human axial high myopia
Source: Cell Res. 2026 Apr 1;36(8):567–81. doi: 10.1038/s41422-026-01241-9 (PMC13424129; doi:10.1038/s41422-026-01241-9)
Supplement: Supplementary file 16 — Supplementary Information, Table S7 [file 41422_2026_1241_MOESM16_ESM.pdf]

Supplementary information, Table S7

Guinea pig–specific α-opic irradiance values.

	Standard White LED	Blue LED (λ_{peak} = 440 nm)
S-cone-opic irradiance ($W \cdot m^{-2}$)	0.10	2.47
M-cone-opic irradiance ($W \cdot m^{-2}$)	0.82	5.91
Rhodopic irradiance ($W \cdot m^{-2}$)	0.74	6.79
